# Liver X receptors activation, through TO901317 binding, reduces neuroinflammation in Parkinson’s disease

**DOI:** 10.1371/journal.pone.0174470

**Published:** 2017-04-03

**Authors:** Irene Paterniti, Michela Campolo, Rosalba Siracusa, Marika Cordaro, Rosanna Di Paola, Vittorio Calabrese, Michele Navarra, Salvatore Cuzzocrea, Emanuela Esposito

**Affiliations:** 1 Department of Chemical, Biological, Pharmaceutical and Environmental Sciences, University of Messina, Messina, Italy; 2 Department of Chemistry, Faculty of Medicine, University of Catania, Catania, Italy; 3 Department of Drug Sciences and Health Products, University of Messina, Messina, Italy; 4 Department of Pharmacological and Physiological Science, Saint Louis University School of Medicine, Saint Louis, Missouri, United States of America; Universita degli Studi di Napoli Federico II, ITALY

## Abstract

Parkinson's disease (PD) is a neurodegenerative disease in which degeneration of nigrostriatal neurons and inflammation are key players. The aim of our study was to analyze the function of LXRs in neurodegenerative diseases as PD using *in vivo*, *ex vivo* and *in vitro* models of PD; for this purpose, we observed the effects of the LXR agonist, TO901317, in neuroinflammatory pathway related to PD. We performed an *in vivo* model of PD using the neurotoxin 1-methyl-4-phenyl-1, 2,3,6-tetrahydropyridine (MPTP) and our results clearly showed that TO901317 administration reduces all of the inflammatory markers involved in PD such as iNOS and COX2, IκB-α and NF-κB. Moreover, to confirm the neuroprotective properties of TO901317, that we obtained with the in vivo model, we performed also an *ex vivo* and *in vitro* models of PD. All the results taken, confirmed that TO901317 is able to modulate the neuroinflammatory pathway involved in PD increasing the locomotors function. Therefore, TO901317, LXR synthetic agonist, could be studied as a new target in a neurodegenerative disorder like PD.

## Introduction

Parkinson`s disease (PD) is the most common neurodegenerative diseases after Alzheimer’s disease (AD), characterized by loss of specific populations of neurons including those in substantia nigra pars compacta (SNpc) and sympathetic ganglia as well as formation of Lewy bodies (LB). LB are eosinophilic citoplasmatic inclusions composed by insoluble aggregates of different proteins, mainly α-synuclein and ubiquitin [1].

The main clinical phenotype of PD are motor symptoms such as: resting tremor, rigidity, bradykinesia (slowness of movements) and impairment of postural instability reflex [2].

Moreover, neuroinflammation plays an important key role in the pathogenesis of PD. For example, some pro-inflammatory cytokines, such as interleukin (IL)-1β, tumor necrosis factor (TNF)-α, and others, can be found at higher levels in cerebrospinal fluid samples of patients affect to PD compared to age-matched controls. Further supporting the involvement of inflammation, activated microglia can be detected in brains from living PD patients and in post-mortem samples from people who is affected of PD [3]. Although treatments to ameliorate clinical appearances of PD are common, there are no pharmacological therapies able to moderate neurodegeneration and death induced by neuroinflammation in PD.

Liver X receptors (LXRs) are involved in the control of inflammatory process in the central nervous system (CNS), in fact previous in vitro studies have shown that LXR agonists attenuate inflammation by inhibiting NF-κB activity, and the expression of inflammatory mediators, such as inducible nitric oxide synthase (iNOS), cyclooxygenase-2 (COX-2), and pro-inflammatory cytokines in microglia and astrocytes [4–6]. Whereas LXR-α is expressed predominantly in liver, kidney, intestine, and tissue macrophages, LXR-β is highly expressed in the brain. The importance of LXR-β in brain function is supported by previous studies showing that LXR-β deficiency is associated with central nervous system pathologies and brain development abnormalities as reported in recent studies in which the genetic ablation of LXRß in APP transgenic mice results in increased amyloid plaque load [7] demonstrating that LXRs ameliorate the pathogenesis of the neurodegenerative diseases, such as AD, PD, multiple sclerosis, and Huntington's disease.

Thus, LXR agonists induce transcriptional activity of LXR target genes, attenuating the astrogliosis and microgliosis induced by inflammation and are widely used in different neurodegeneration animal models [8, 9]. Based on our previous studies, in which we demonstrated the important involvement of TO901317 on the modulation of neuroinflammation associated with spinal cord trauma [4], here we concentrated on the function of LXRs in regulating neuroinflammation related to PD using *in vitro*, *ex vivo* and *in vivo* models of PD, with particular attention on current advances in the understanding of the potential therapeutic role of LXR agonist.

## Materials and methods

### Animals

C57/BL6 mice (male 25–30 g, 7 weeks age old; Envigo, Italy) were housed in a controlled environment and provided with standard rodent chow and water. Animal care was in accordance with Italian regulations on defence of animals used for experimental and other scientific purposes (D.M. 116/92) as well as with the EEC regulations (O.J. of E.C. L 358/1 12/18/1986). In particular this study was approved by the animal ethics board of the University of Messina in 12^th^ October 2012, in agreement with 3 comma 1 and 4 del D.L. 116/92.

### MPTP-induced Parkinson’s disease and treatment

Eight-week-old male C57 mice received four intraperitoneal injections of 1-methyl-4-phenyl-1,2,3,6-tetrahydropyridine (MPTP) (20 mg/kg; Sigma, St. Louis, MO) in saline at 2hr intervals in 1 day, the total dose per mouse is 80 mg/kg. Starting 24 hours after the first MPTP injection and continuing through 7 additional days after the last injection of MPTP, mice were treated with TO901317 (20 mg/kg in 10% DMSO formulation). Animals were sacrificed 8 days after MPTP injection and their brains were harvested, sectioned and processed.

The dose of MPTP (80 mg/kg.) and TO901317 (20 mg/kg) used here were based on our precedent *in vivo* studies [10, 11]

No one of the animals utilized in this study didn’t become ill or died prior to the experimental endpoint.

### Experimental groups

Mice were arbitrarily allocated into the following groups.

Group 1: Sham+Veh = vehicle solution (saline) was administered during the 1^st^ day, like MPTP protocol, intraperitoneally. (N = 10)

Group 2: Sham+TO901317 = same as the Sham+Veh group, but TO901317 (20 mg/kg body in 10% DMSO formulation i.p.) was administrered starting 24hrs after the first vehicle solution injection and continuing through 7 additional days after the last injection of saline. (N = 10)

Group 3: MPTP+Veh = MPTP solution was administered as described before plus administration of saline. (N = 10)

Group 4: MPTP+TO901317 = same as the MPTP+Veh group, but TO901317 (20 mg/kg body in 10% DMSO formulation i.p.) was administrered starting 24 hrs after the first MPTP injection and continuing through 7 additional days after the last injection of MPTP. (N = 10)

### Behavioural testing

Behavioural assessments on each mouse were made 1 day prior to, and 8 days after, MPTP injection:

#### Force swim test (FST)

This test was made as previously indicated by Porsolt et al. [12]. All mice were gently placed in the cylinder for 6 minutes and the duration of floating was scored. Immobility time was analyzed during the last 4 min period of the test.

#### Elevated plus-maze test (EPM)

The elevated plus-maze protocol was performed as previously described [13, 14].

#### Catalepsy test

The catalepsy test was evaluated at 8 days after MPTP injection, as previously observed [15].

### Immunohistochemical localization of TH, DAT, COX-2, iNOS, BAX and Bcl-2

The animals were anesthetized with ketamine and xylazine (2.6 and 0.16 mg/kg body weight respectively) 8 days after MPTP intoxication. The brain sections were processed as previously explained [10] and incubated overnight with anti-TH polyclonal antibody (Millipore 1/500 v/v), anti-DAT antibody (Santa Cruz Biotechnology sc14002, 1/300 v/v), anti-COX-2 antibody (Cell signalling, 1/250 v/v), anti-iNOS antibody (BD Transduction, 1/500 v/v), anti-Bax antibody (Santa Cruz Biotechnology, 1/100 in PBS, v/v) and anti-Bcl-2 antibody (Santa Cruz Biotechnology, 1/500 v/v). Immunohistochemical images were collected using a Zeiss microscope and Axio Vision software. For graphic display of densitometric analyses, the intensity of positive staining (brown staining) was measured by computer-assisted color image analysis (Leica QWin V3, UK). The percentage area of immunoreactivity (determined by the number of positive pixels) was expressed as per cent of total tissue area (red staining). Replicates for every experimental condition and histochemical staining were acquired from each mouse in all experimental group.

### Cytosolic and nuclear extracts from midbrain and western blot analysis

Tissue samples from the brain were processed in brief the expression of Glial fibrillary acidic protein (GFAP) and IBA1 was quantified in cytosolic fraction from brain tissues. The filters were blocked with 1 × PBS, 5% (w/v) non fat dried milk (PM) for 1 h at room temperature and then probed with specific Abs anti-GFAP (1/500; Santa Cruz Biotechnology) or anti-IBA1 (1/500; Santa Cruz Biotechnology), in 1 × PBS, 5% w/v non fat dried milk, 0.1% Tween-20 (PMT) at 4°C, overnight. Signals were detected with enhanced chemiluminescence (ECL) detection system reagent and the relative expression of the protein bands was quantified by densitometry with BIORAD ChemiDoc^TM^XRS+software, standardized to β-actin and lamin A/C levels, as previously described [10].

### Preparation of organotypic cultures from the ventral mesencephalon

Ventral mesencephalon slice cultures were prepared from mouse brain at postnatal day 6, as previously described [11]. Organotypic cultures were examined on a daily basis to observe general structural integrity.

After 7 days of stabilization, organotypic slice cultures were subdivided into 3 groups:

1. Control (CTR): intact ventral mesencephalon (VM) slices were cultured with normal culture medium.
2. MPTP: the VM slices were stimulated with MPTP 50 μM.
3. MPTP+TO901317: the VM slices were stimulated as previously described and TO901317 (10 μM) was applied 2 hrs before injury. This drug was left in a culture medium for 24 hours after injury.

The dose of MPTP (50 μM) used here was based on previous dose–response and time-course studies by our laboratory.

### Cell culture

For the *in vitro* model, we used human neuronal cells SH-SY5Y cells (ATCC CLR-2266), a sub clone derived from the human neuroblastoma cell line SK-N-SH originated from a tumor in the bone metastasis. The cells were tested for contamination before use. These well-differentiated cells displays typical dopaminergic characteristics, including expression of tyrosine hydroxylase, dopamine transporter and monoamine oxidase A & B, making this a typical model for dopaminergic neurons [16]. SH-SY5Y neuroblastoma cells can be differentiated into sustainable neuronal morphology. We found the best overall neuronal differentiation was obtained using RA pretreated SH-SY5Y cells.

A preliminary analysis involved the study of cell viability; 3x10^4^ cells was plated in a volume of 150 μl in 96-well plates and differentiated with retinoic acid (100 nM) for 24 hours. After 24 hours of differentiation increasing concentrations of TO901317 (0,001–100 μM) were used to determine the effective concentration with minimal toxic effect on cell viability.

In another set of experiments, 3x10^4^ cells were plated and differentiated as all the other set of experiment for 24 hours. After differentiation, cells were pre-treated for 2 hours with TO901317 at the concentration of 10 μM (based on previous MTT assay) and stimulated with 1-methyl 4-phenyl 1,2,3,6-tetrahydro-pyridine (MPTP) (3 mM).

Moreover, 8x10^5^ cells were plated and differentiated as all the other set of experiment to performed western blot analysis. After 24 hours cells were lysates.

SH-SY5Y cultures were divided into 4 groups:

1. Control group (CTR): differentiated cells were cultured with normal medium.
2. Control+TO901317 (CTR+TO901317): differentiated cells were stimulated with TO901317 (10 μM);
3. MPTP group: differentiated cells were stimulated with MPTP (3 mM);
4. MPTP+TO901317: differentiated cells were stimulated with MPTP (3 mM) and TO901317 (10 μM) 2 hour before stimulation.

The dose of MPTP (3 mM) used here was based on precedent *in vitro* studies [17].

### Vital staining

To assess the viability of cell cultures, 3x10^4^ cells was plated in a volume of 150 μl in 96-well plates and differentiated with RA. Increasing concentrations of TO901317 (0,001–100 μM) were used to determine the effective concentration with minimal toxic effect on cell viability. Cells were incubated with and without MPTP stimulation. At 24 hours viability of cell cultures was assessed using a mitochondria-dependent dye for live cells (tetrazolium dye; MTT) to formazan, as previously indicated [18].

### Western blot analysis

SH-SY5Y cells and VM organotypic cultures were treated with a lysis buffer and protein concentrations were estimated by the Bio-Rad protein assay using bovine serum albumin as standard, as previously described [10]. Specific primary antibody anti-iNOS (BD Transduction 610329; 1/500), anti-COX2 (Cell Signaling #4842 1/500), anti-Bid (1/500; Santa Cruz Biotechnology), anti-Bad (1/500; Santa Cruz Biotechnology), anti-IκBα (Santa Cruz Biotechnology sc-371; 1/500), anti-NFκB p65 (Santa Cruz Biotechnology sc-372; 1/500), anti-Bcl2 (Santa Cruz Biotechnology sc-7382; 1/500) and anti-Bax (Santa Cruz Biotechnology sc-7480; 1/500) were mixed in 1× PBS, 5% w/v nonfat dried milk, 0.1% Tween-20 (PMT) and incubated at 4°C, overnight. Signals were detected as described above [11].

### Materials

TO901317 was obteined from Tocris Bioscience, all other compounds were obtained from Sigma-Aldrich Company Ltd. (Milan, Italy). All stock solutions were made in non-pyrogenic saline (0.9% NaCl; Baxter, Italy, UK).

### Statistical evaluation

All values in the figures and text are expressed as mean ± standard error of the mean (SEM) of N observations. The figures are representative of the three experiments performed on different experimental days. The western blots analyses are representative of 3 different gels made by dividing the number of samples obtained from 10 animals for each experimental group in different days. For *in vivo* studies, N represents the number of animals studied. The results were analyzed by one-way ANOVA followed by a Bonferroni post-hoc test for multiple comparisons. A p-value of less than 0.05 was considered important. The minimum number of animals or samples for each technique was calculated with the statistical test *a priori* power analyzes of the G-power software, this statistical test provides an efficient method to determine the sample size necessary to perform the experiment.

## Results

### Effect of TO901317 on behavioural impairments induced by MPTP intoxication

**Fig 1:**
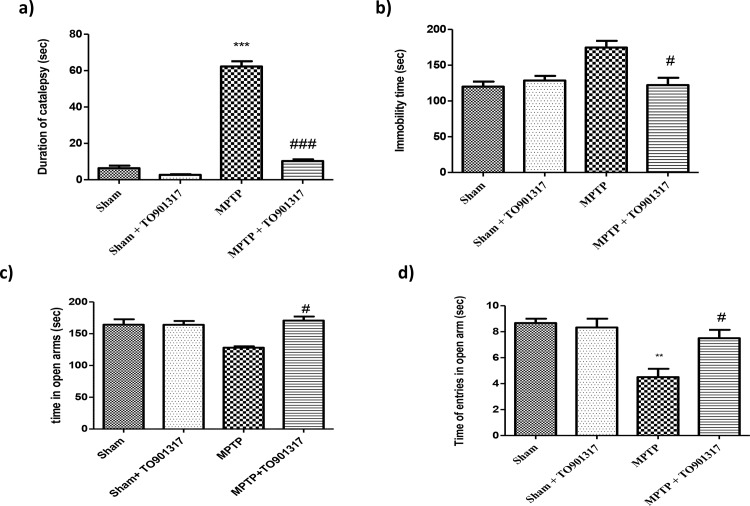
Role of TO901317 on motor deficit induced by MPTP. Mice were analysed for motor deficit associated to PD with the catalepsy test. We observed that MPTP induced a major motor deficit respect to sham group, whereas TO901317 enhanced motor deficit (a). Moreover, mice were tested for depression-like behaviour in the FST (b). Bar graphs show an increases in the immobility time(s) of MPTP-lesioned mice, while TO901317 treatment significantly reduce immobility time (b). Results are expressed as mean of mobility duration in seconds. Values are mean ± SEM (N = 10 animals per group). ***p < 0.001 vs Sham; ## p < 0.01 vs MPTP (a). To test anxiety we performed the elevated plus maze test. Anxiety is evinced by the mean total access in the open arms and number of entries in open arms (c and d respectively) that were significantly enhanced in TO901317 treatment group, compared to MPTP group. Values are mean ± SEM (N = 10 animals per group). **p < 0.01 vs Sham; # p <0.05 vs MPTP.

To evaluate the effect of TO901317 on PD-associated motor deficits produced by MPTP lesion, we executed catalepsy test in which MPTP administration produced a important increases in the motor deficit and in the cataleptic response. The MPTP-induced motor failures were significantly reduced by TO901317 treatment (Fig 1A). The effect produced by MPTP on depressive-like behaviour was also examined in the forced swim test. We found that, in the FST, MPTP- lesioned mice displayed a longer immobility time (considered an index of depression) in comparison to sham mice. While TO901317 enhanced MPTP-induced immobility (Fig 1B). Moreover, mice were then examined for anxiety-like behaviour in the elevated plus maze test. The behavioural test indicated an important increase in the percentage of time passed in the open arms and in the total entrance in the open arms after TO901317 treatment, compared to the MPTP group, hereby indicating an antidepressant-like effect of TO901317 (Fig 1C and 1D).

### TO901317 treatment reduced the loss of TH and DAT expression in the SN induced by MPTP administration

To confirm the results obtained, we wanted to evaluate specific markers of PD such as Tyrosine hydroxylase (TH) and Anti-Dopamine Transporter (DAT).

**Fig 2:**
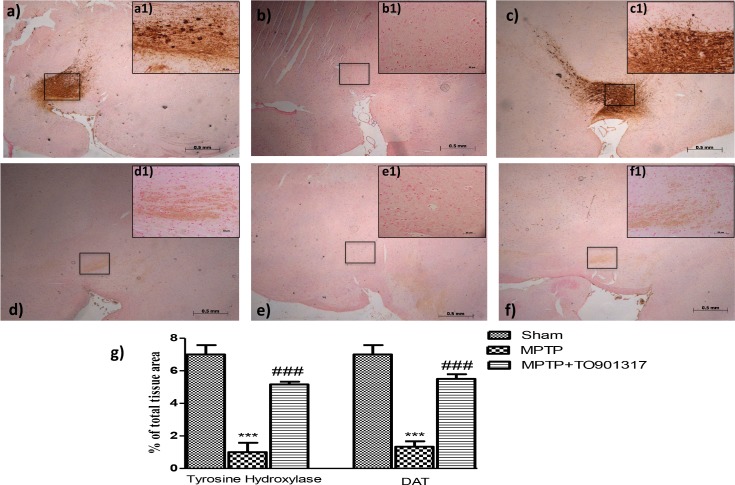
Effects of LXR agonist on specific marker of PD. An important reduction of TH activity was observed in MPTP-group (b, b1 see densitometry analysis g, and Figure b in S1 File.), whereas the TO901317 treatment restored the loss of TH-positive neurons S (c,c1 see densitometry analysis g and Figure c in S1 File). Moreover, MPTP administration produced an important reduction of DAT expression (e, e1, see densitometry analysis in g, and Figure b in S2 File) that was enhanced by TO901317 treatment (f, f1. see densitometry analysis g, and Figure c in S2 File). Values are mean ± SEM (N = 10 animals per group). ***p < 0.001 vs Sham; ### p <0.001 vs MPTP. For the magnification scale 2.5 x, scale bar is 0,5mm (a,b,c,d,f,e); for the magnification scale 20x, scale bar is 50 μm (a1,b1,c1,d1,f1,e1).

In the SN an important loss of TH-positive cells was observed in MPTP-injected animals (Fig 2B, see particle b1 and relative densitometry analysis g), while the treatment with TO901317significantly reduced the loss of TH-positive neurons in the SN (Fig 2C, see particle c1and relative densitometry analysis g). Moreover, to analyze the effects of LXR agonist treatment on the dopaminergic pathway we also evaluated, by immunohistochemistry, the levels of DAT. We observed an important loss of DAT in MPTP group (Fig 2E, see particle e1 and relative densitometry analysis g) whereas TO901317 treatment significantly restored the levels of DAT comparable to control group (Fig 2F, see particle f1 and relative densitometry analysis 2g).

### Role of TO901317 on iNOS and COX-2 expression as well as on astrocytes and microglia activation following MPTP

**Fig 3:**
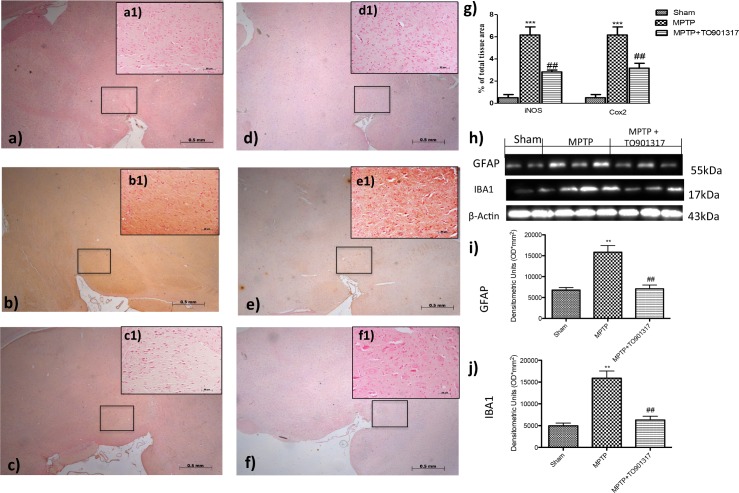
Effect of TO901317 on GFAP, Iba1, iNOS and COX-2 expressions. GFAP and Iba1 were detected by western blot analysis, to provide a quantitative estimation of astrocytes and microglial activation. GFAP and Iba1 expression increased significantly after MPTP treatment (h, i, j and Figure a in S7 File), instead treatment with TO901317 significantly reduced this expressions (h, i, j and Figure a in S7 File). i and j ** p <0.01 vs Ctr; ## p <0.01 vs MPTP.A clear positive staining for iNOS and COX2 was found in MPTP group (b, b1, e,e1, see densitometry analysis g, Figure b in S3 File and Figure b in S4 File), whereas treatment with TO901317 noticeably prevented enhanced expression of iNOS and COX2 (c,c1, f,f1,. see densitometry analysis g, and Figure c in S3 File and Figure c in S4 File). Values are mean ± SEM (N = 10 animals per group). ***p < 0.001 vs Sham; ## p <0.01 vs MPTP. For the magnification scale 2.5 X, scale bar is 0,5mm (a,b,c,d,f,e); for the magnification scale 20X, scale bar is 50 μm (a1,b1,c1,d1,f1,e1).

To determine the role of nitric oxide (NO) in the development of PD, iNOS expression was evaluated by immunohistochemistry staining. A marked positive immunostaining for iNOS was found after MPTP administration (Fig 3B, see particle b1and densitometry analysis g); instead treatment with TO901317 prevented the enhanced expression of iNOS immunoreactive cells in the SN (Fig 3C, see particle c1and relative densitometry analysis g).

Furthermore, we also evaluated the expression of COX-2, to determine the effects of MPTP on lipid degradation and the subsequent production of leukotriene and prostaglandins. By immunohistochemistry staining we observed a significantly increase in COX-2 expression in MPTP group (Fig 3E, see particle e1and relative densitometry analysis g); instead TO901317 treatment prevented the enhanced appearance of COX-2 in immunoreactive cells in the SN (Fig 3F, see particle e1and relative densitometry analysis g).

To better investigate if LXR agonist could modulate neuroinflammation reducing the degree of reactive astrocytosis and microgliosis, we performed western blot analysis for GFAP (a marker of activated astrocytes) and Iba1 (a marker for microglia) [19].

The results obtained showed that a greater astroglial reaction was evident after MPTP administration as well as Iba1 expressions (Fig 3H, 3I and 3J); while treatment with TO901317 was able to reduce significantly the activation of GFAP and Iba1 (Fig 3H, 3I and 3J).

### Effect of LXR agonist treatment on the alteration of Bax and Bcl-2 expression after MPTP intoxication

To understand if MPTP treatment was associated with cell death, we evaluated the specific marker of apoptosis such as Bax and Bcl-2.

**Fig 4:**
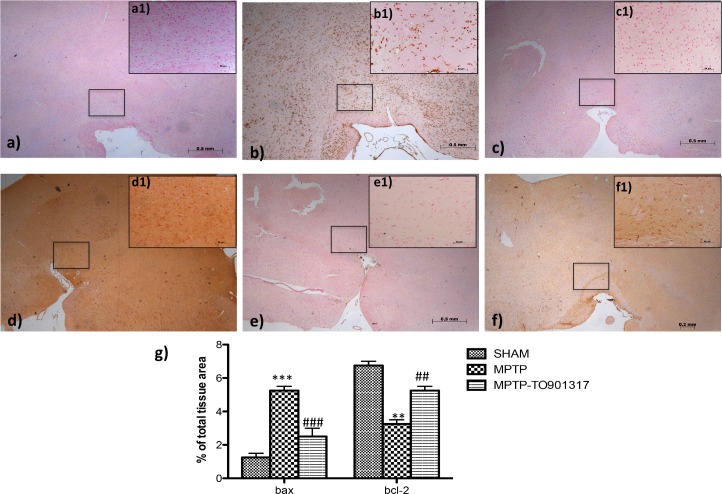
Effects of TO901317 on apoptosis. The results underlined that Bax expression was considerably enhanced in the MPTP group (b, b1 see densitometry analysis g, and Figure b in S5 File) whereas treatment with TO901317 significantly limited the rise in Bax expression (c,c1 see densitometry analysis g and Figure c in S5 File). In addition, a marked positive staining for Bcl-2 was found in control and treated mice (d,d1, f,f1, respectively, see densitometry analysis g, and Figure a and c in S6 File); whereas MPTP induced a decreased expression of Bcl-2 (e,e1 see densitometry analysis g and Figure b in S6 File). Values are mean ± SEM (N = 10 animals per group) *** p < 0.001 vs Sham; ### p < 0.001 vs MPTP; ** p < 0.01 vs Sham; ### p < 0.01 vs MPTP. For the magnification scale 2.5 X, scale bar is 0,5mm (a,b,c,d,f,e); for the magnification scale 20 X, scale bar is 50 μm (a1,b1,c1,d1,f1,e1).

Immunohistochemistry analysis showed that Bax levels was significantly enhanced after MPTP administration (Fig 4B, see particle b1and relative densitometry analysis g); while treatment with LXR agonist clearly restored the expression of Bax (Fig 4C see particle c1and relative densitometric analysis g).

Anti-apoptotic factor, such as Bcl-2, was also investigated by immunohistochemistry. The results obtained demonstrated a marked positive immunostaining for Bcl-2 after TO901317 administration (Fig 4F see particle f1and relative densitometry analysis g); instead in MPTP-treated mice we showed a significantly reduction of Bcl-2 immunoreactive cells in the SN (Fig 4E, see particle e1and relative densitometry analysis g) compared to the control group.

### Effect of TO901317 on expression of pro-inflammatory enzymes in organotypic cultures of ventral mesencephalon

**Fig 5:**
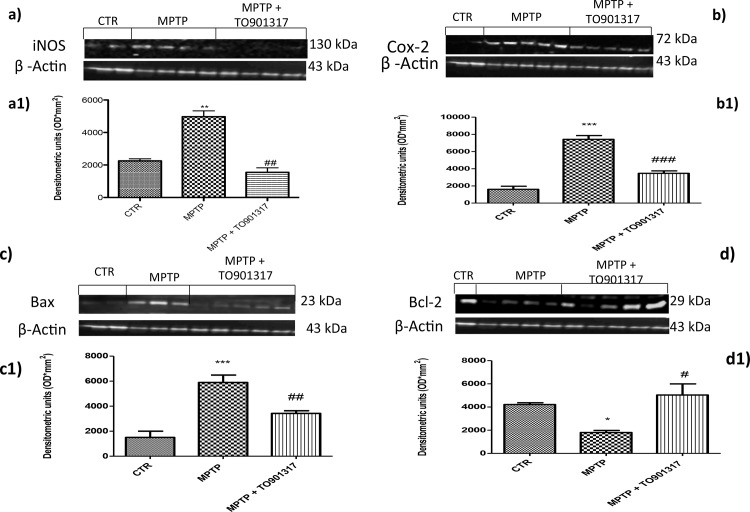
Expression of iNOS, COX-2 and apoptotic levels in organotypic cultures of ventral mesencephalon. An important increase in iNOS expression in the organotypic cultures was observed in MPTP group (panels a and a1), whereas TO901317 treatment significantly lowered the expression of iNOS (panels a and a1). Furthermore, an important enhanced expression of COX-2 was observed in the MPTP group (panels b,b1), expression that was significantly decreased by TO901317 treatment (panels b and b1). Values are mean ± SEM (N = 10 animals per group). a1) ** p < 0.01 vs Sham; ## p < 0.01 vs MPTP; b1) *** p < 0.001 vs Sham; ### p < 0.001 vs MPTP. Moreover, the apoptotic marker such as Bax was significantly enhanced in the MPTP group (panels c,c1), expression that was clearly decreased with TO901317 (panels c and c1). In addition, Bcl-2 expression was lowered after MPTP stimulation (panels d,d1); while the treatment with TO901317 significantly restored basal levels of Bcl-2 (panels d and d1). The results obtained confirmed the previous in vivo observed results. Values are mean ± SEM (N = 10 animals per group). c1)*** p < 0.001 vs Sham; ## p < 0.01 vs MPTP; d1) * p < 0.05 vs Sham; # p < 0.05 vs MPTP.

To better investigate the role of the nitrosative stress in the development of PD we evaluated the enhanced expression of iNOS in an *ex vivo* model of organotypic cultures. By western blot analysis, basal levels of iNOS were observed in the controls group, instead MPTP administration induced an significantly enhanced iNOS expression (Fig 5A and 5A1). The pre-treatment by TO901317 clearly restored the expression of iNOS (Fig 5A and 5A1). Furthermore, we would confirm the expression of COX-2 observed in vivo. The levels of COX-2 were significantly enhanced after stimulation with MPTP, while a reduction of COX-2 levels was evident after pre-treatment with TO901317 (Fig 5B and 5B1).

### Effect of TO901317 on expression of apoptotic proteins in organotypic cultures of ventral mesencephalon

To better understand the involvement of apoptosis in PD we also evaluated, by western blot analysis, the levels of Bax and Bcl-2 in the organotypic cultures. We observed that Bax levels were significantly enhanced after stimulation with MPTP, while pre-treatment with TO901317 significantly reduced Bax levels confirming the in vivo results (Fig 5C and 5C1). Moreover, we demonstrated that the basal levels of Bcl-2 were reduced after MPTP exposure while pre-treatment with TO901317 clearly restored Bcl-2 levels (Fig 5C and 5C1.).

### Effect of TO901317 on expression of IκB-α and nuclear translocation of NF-κB p65 in SH-SY5Y cell cultures

**Fig 6:**
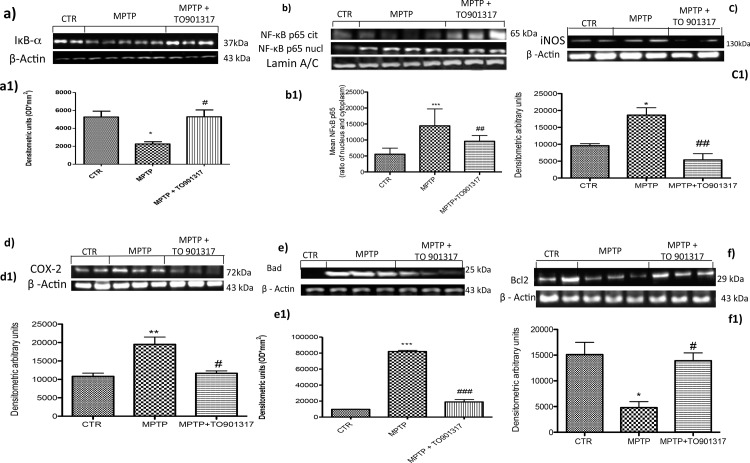
Effect of TO901317 on inflammatory process and apoptotic event in SH-SY5Y cell cultures. TO901317 treatment decrease IκBα degradation (panels a, a1 and Figure b in S7 File) and decreased p65 nuclear translocation (panels b, b1) induced by MPTP exposition. In addition we observed that TO901317 treatment modulate the cytoplasmic rate of NFκB as showed in panels b and b1. a1)* p <0.05 vs Ctr; # p <0.05 vs MPTP; b1) *** p <0.001 vs Ctr; ## p <0.01 vs MPTP. Moreover, we observed that TO901317 treatment significantly lowered the expression of iNOS and COX-2 (panels c,c1, d,d1 and Figure c and d in S7 File). * p < 0.05 vs Sham; ## p < 0.01 vs MPTP; d1) ** p < 0.01 vs Sham; # p < 0.05 vs MPTP. SH-SY5Y lysates showed how the levels of Bad (e, e1) enhanced significantly after MPTP exposure, instead pre-treatment with TO901317 clearly reduced Bad expression (e,e1). Moreover, the levels of Bcl-2 were significantly restored after the treatment with TO901317 (panels f,f1). e1) ***p <0.001 vs Ctr; ###p < 0.001 vs MPTP; f1) * p <0.05 vs Ctr; # p <0.05 vs MPTP.

To investigate the cellular mechanism through TO901317 could attenuate inflammatory processes induced by MPTP, we assessed in *in vitro* model of PD, the degradation of IκB-α and the nuclear translocation of NF-κB p65 factor. The results obtained by Western Blot analysis, showed a basal expression of IκB-α in the cytoplasmic fraction of control cells, while IκB-α levels decreased significantly after stimulation with MPTP. Pre-treatment with TO901317significantly inhibited the degradation of IκB-α bringing its expression at levels comparable to value of the control group (Fig 6A and 6A1). Moreover, p65 subunit translocation protein were enhanced after MPTP exposition, instead pre-treatment with TO901317 significantly reduced NF-κB levels (Fig 6B and 6B1). To confirm that the LXR agonist significantly reduced p65 nuclear translocation, we observed by western blot the rate of p65 translocation from the cytoplasm to the nucleus, analysing the citoplasmatic NF-κB levels (Fig 6B and 6B1). Nuclear translocation of NF-κB p65 in turn activated a cascade of inflammation mediators such as iNOS and COX-2, that are significantly activated by MPTP stimulation (Fig 6C, 6C1, 6D and 6D1 respectively), whereasTO901317 reduced the expression of both iNOS and COX-2 (Fig 6C, 6C1, 6D and 6D1).

### Effect of TO901317 on the apoptotic process in SH-SY5Y cell cultures

In order to investigate the role of LXR on apoptotic events induced by MPTP we assessed, by western blot analysis, the expression of pro-apoptotic protein such as Bad. The results obtained showed an important increase of Bad levels after MPTP exposure, while a reduction of expression of Bad was evident after pre-treatment with TO901317 (Fig 6E and 6E1). Moreover we analysed the anti-apoptotic factor Bcl-2 and we observed the MPTP significantly reduced the expression of Bcl-2; instead TO901317 treatment significantly restored Bcl-2 expression (Fig 6F and 6F1), confirming the results obtained in *in vivo* model of PD.

## Discussion

Parkinsonism is most common chronic neurodegenerative disorders that affect motor coordination. Although the pathophysiology of PD is not well understood, the actually pharmacological treatment is direct primarily to elevate striatal DA levels or deep-brain electrical stimulation.

Numerous efforts have been done to find neuroprotective agents for PD and one of this is the use of a valid animal model that mimic the pathogenesis of PD such as MPTP animal model, that is efficacy to understand the neurodegeneration in PD because it produces clinical, biochemical and neuropathological alterations comparable to those identified in human [20]. The liver X receptors (LXR α (NR1H3) and LXR β (NR1H2)), members of the nuclear receptor superfamily, control numerous of physiological processes such as cholesterol metabolism and transport, lipogenesis, gluconeogenesis, and inflammation. Synthetic LXR agonists demonstrated an important role in disorders such as dyslipidemia, atherosclerosis, and diabetes.

Moreover, previous research has demonstrated lipid deposition, gliosis and degeneration of cells located in the substantia nigra of aged LXR double-knockout animals [7], whereas LXRβ−/− mice develop ALS–Parkinson-like syndrome after 6 months of age.

These observations suggest that LXRβ and no FXR may be protective against neurodegeneration of substantia nigra [21]. Thus, here we sought to observe whether LXR agonist has the ability to ameliorate the pathological symptoms generated in an MPTP mouse model of PD. Here, animals were administered with TO901317, a potent LXR receptor ligand that show equal affinity for both LXR receptors (α and β). We used TO901317 to explore whether it could protect the animals from the toxicity and neuroinflammation generated by MPTP treatment and if prevent or attenuate vascular damage in the caudate putamen (CPu) and SNc that is often seen in PD patients. Further, we analyzed the properties of LXR agonist in *an ex vivo* model of PD using organotypic cultures from the ventral mesencephalon and an *in vitro* model using SH-SY5Y cell cultures.

In this study, first we observed the alteration of the specific marker of PD such as TH, that is the enzyme responsible for catalysing the conversion of L-tyrosine to dihydroxyphenylalanine (DOPA), a precursor for dopamine, and of DAT a member of a large family of Na+-Cl−lCember of aopamine transporters. We observed that TO901317 significantly restored the levels of TH and DAT in the CPu and SNc in mice treated chronically with MPTP, that is correlated with the ability of TO901317 to prevent the deterioration of DA neurons in this mouse model of PD.

Moreover, it has been known that MPTP affecting dopaminergic neurons causes important decrease on locomotor activity. To study the motor behavioral functions we performed the FST and catalepsy tests that are considered the most sensitive tests to assess motor function mice. In this study, we demonstrated that TO901317 significantly improved latency versus MPTP groups, indicating that LXR agonist mechanisms promote recovery and enhance repair mechanism.

One of the key features of PD pathology is neuroinflammation and it is now recognized that targeting neuroinflammation is one intervention that can slow down the development of PD [3, 22, 23].

In the current work we observed that TO901317 treatment significantly reduced the enhanced expression of microgliosis and astrogliosis respectively observed by IBA-1 and GFAP activation.

Activation of microglia determinate the release of a series of pro-inflammatory and neurotoxic proteins such as TNF-α, IL-1β and free radicals formation, all of which can disrupt the BBB [24, 25] that may determinate PD.

Moreover, pro-inflammatory cytokines can stimulate the inducible form of nitric oxide synthase (iNOS) [26–28] and cyclooxygenase 2 (COX2) enzymes that produce toxic reactive species resulting from microglia activation [29]. In support of this finding, our results showed a notable increase of iNOS and COX-2 both *in vivo* and *in vitro* model of PD, while treatment with TO901317 significantly reduced their expression; this is strictly correlated to the production of ROS, a consequence of microglia activation in parkinsonian patients [30].

Moreover, apoptosis is a well-organized cellular process that is involved in death dopaminergic neurons. Although there is more than one pathway to induce apoptosis, the interaction between pro-apoptotic Bax and anti-apoptotic Bcl-2 may determine the outcome of the cell by regulation via the mitochondrial membrane and release of cytochrome-c (cyt c) from mitochondria [31]. Our results indicate that MPTP injection induce the increase levels of Bax and in contrast reduce Bcl-2 levels. The administration of TO901317 significantly enhanced Bcl-2 levels preventing the death of dopaminergic neurons decreasing pro-apoptotic Bax protein, as the ratio of Blc-2/Bax is important in apoptosis [32].

To study the mechanism(s) by which TO901317 exerts these neuroprotective effects, we perform the cellular culture model of PD using SH-SY5Y cell line in which LXR were expressed. As we know pro-inflammatory markers, such as iNOS and COX2, are regulated by Nuclear factor kappa-B protein (NF-κB), we analysed NF-κB levels after MPTP stimulation. We observed that pre-treatment with TO901317 significantly reduced NF-κB p65 and prevented IκBα degradation in SH-SY5Y in vitro model.

Decreasing NF-κB expression TO901317 is able to reduce iNOS and COX2 expression induced after MPTP stimulation and the apoptotic pathway.

However, despite a lot of progress made to understand the pathogenesis of PD, one question remains: are neuroinflammation mechanisms the main cause of the progressive damage of dopaminergic neurons? Our results clearly show that TO901317, a synthetic LXR agonist, is able to modulate the neuroinflammatory pathway involved in PD and can also ameliorated motor function. Therefore, TO901317, LXR synthetic agonist, could be studied as a possible pharmacological target in a neurodegenerative disorders like PD.

## Supporting information

S1 File **a** Original immunohistochemical images for TH for Sham group (magnification scale 2.5 x) **b** Original immunohistochemical images for TH for MPTP group (magnification scale 2.5 x) **c.** Original immunohistochemical images for TH for MPTP-TO901317 group (magnification scale 2.5 x).
(PDF)

S2 File **a** Original immunohistochemical images for DAT for Sham group (magnification scale 2.5 x) **b** Original immunohistochemical images for DAT for MPTP group (magnification scale 2.5 x) **c** Original immunohistochemical images for DAT for MPTP-TO901317 group (magnification scale 2.5 x).
(PDF)

S3 File **a** Original immunohistochemical images for iNOS for Sham group (magnification scale 2.5 x) **b** Original immunohistochemical images for iNOS for MPTP group (magnification scale 2.5 x) **c.** Original immunohistochemical images for iNOS for MPTP-TO901317 group (magnification scale 2.5 x).
(PDF)

S4 File **a** Original immunohistochemical images for COX-2 for Sham group (magnification scale 2.5 x) **b** Original immunohistochemical images for COX-2 for MPTP group (magnification scale 2.5 x) **c** Original immunohistochemical images for COX-2 for MPTP-TO901317 group (magnification scale 2.5 x).
(PDF)

S5 File **a** Original immunohistochemical images for BAX for Sham group (magnification scale 2.5 x) **b** Original immunohistochemical images for BAX for MPTP group (magnification scale 2.5 x) **c** Original immunohistochemical images for BAX for MPTP-TO901317 group (magnification scale 2.5 x).
(PDF)

S6 File **a** Original immunohistochemical images for Bcl-2 for Sham group (magnification scale 2.5 x) **b** Original immunohistochemical images for Bcl-2 for MPTP group (magnification scale 2.5 x) **c** Original immunohistochemical images for Bcl-2 for MPTP-TO901317 group (magnification scale 2.5 x).
(PDF)

S7 File Original western blot images for GFAP, IκBα, COX-2, iNOS. (PDF)
