# Supplementary figures and images for: Liver X receptors activation, through TO901317 binding, reduces neuroinflammation in Parkinson’s disease
Source: PLoS One. 2017 Apr 3;12(4):e0174470. doi: 10.1371/journal.pone.0174470 (PMC5378346; doi:10.1371/journal.pone.0174470)

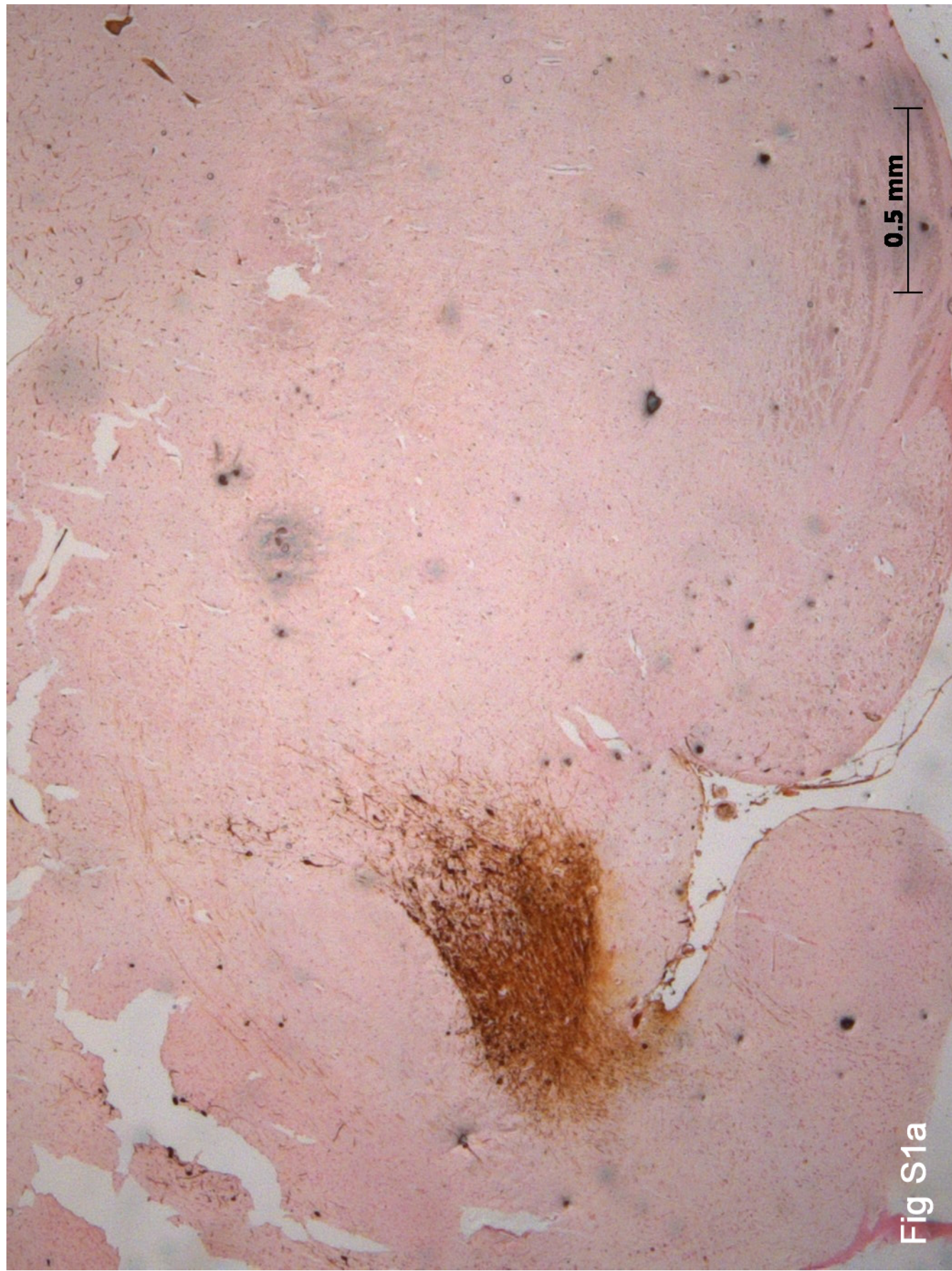

0.5 mm

Fig S1a

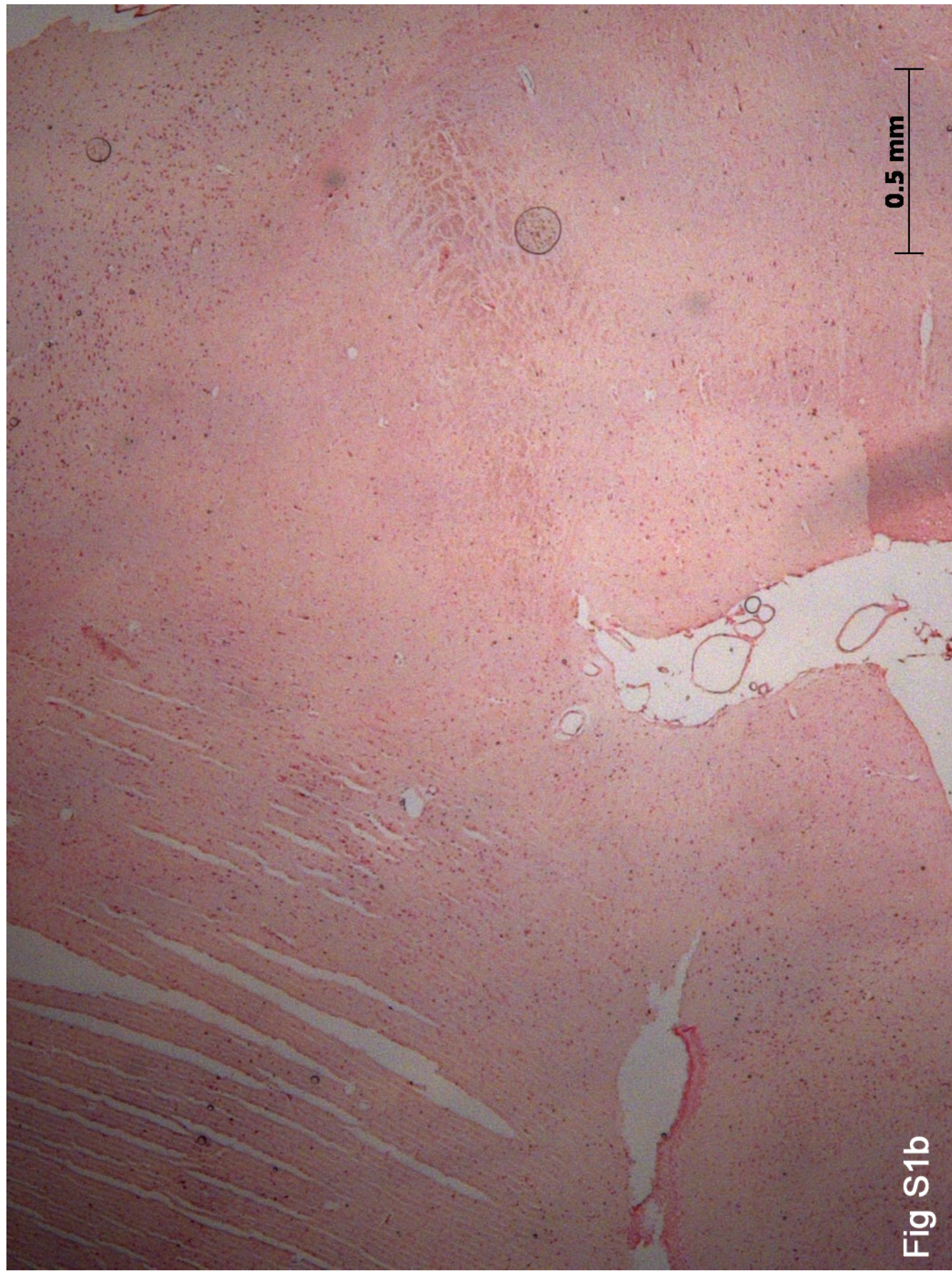

Fig S1b

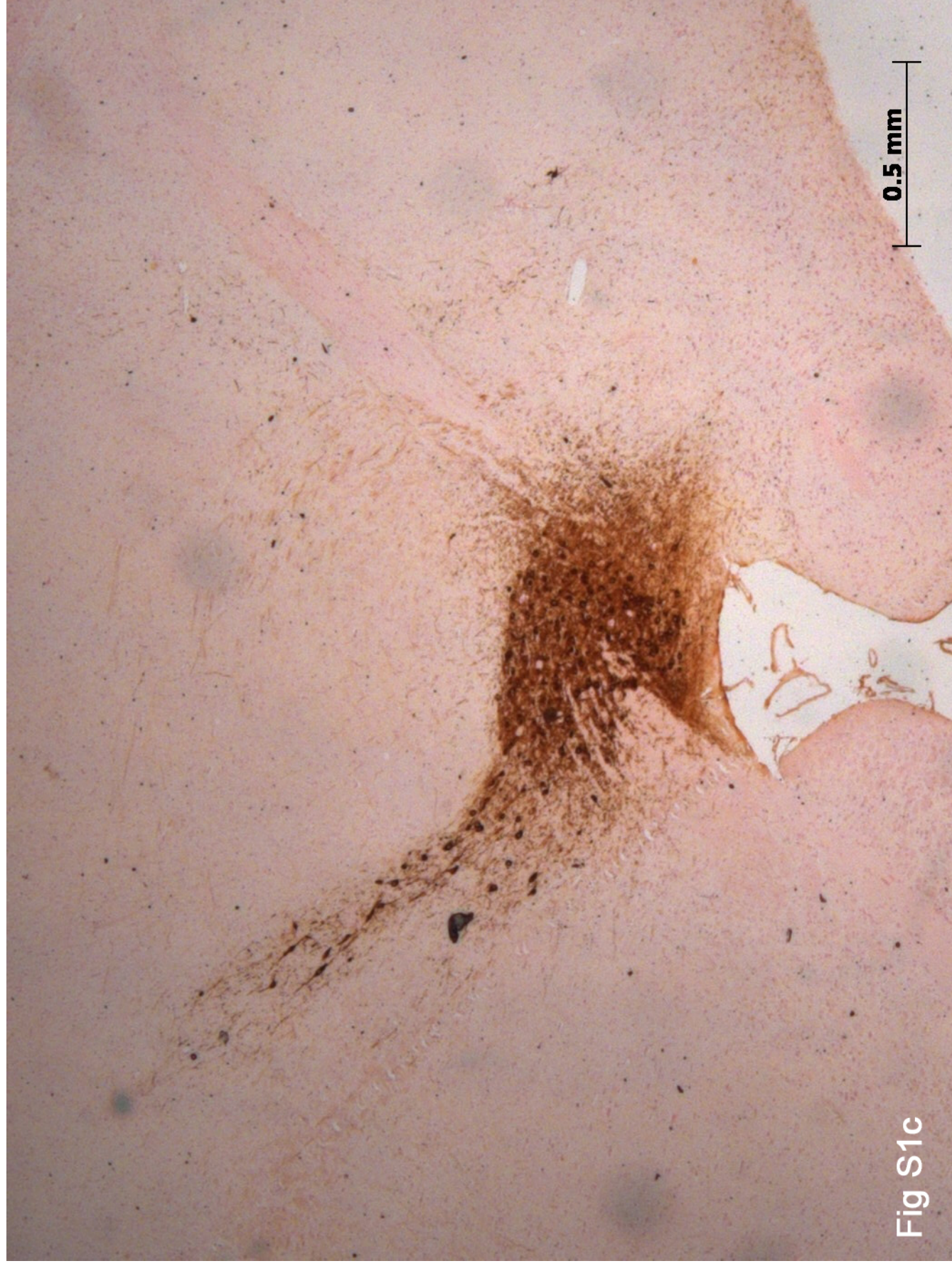

Fig S1c

Supplement: S1 File — a Original immunohistochemical images for TH for Sham group (magnification scale 2.5 x) b Original immunohistochemical images for TH for MPTP group (magnification scale 2.5 x) c. Original immunohistochemical images for TH for MPTP-TO901317 group (magnification scale 2.5 x). (PDF) [file pone.0174470.s001.pdf]

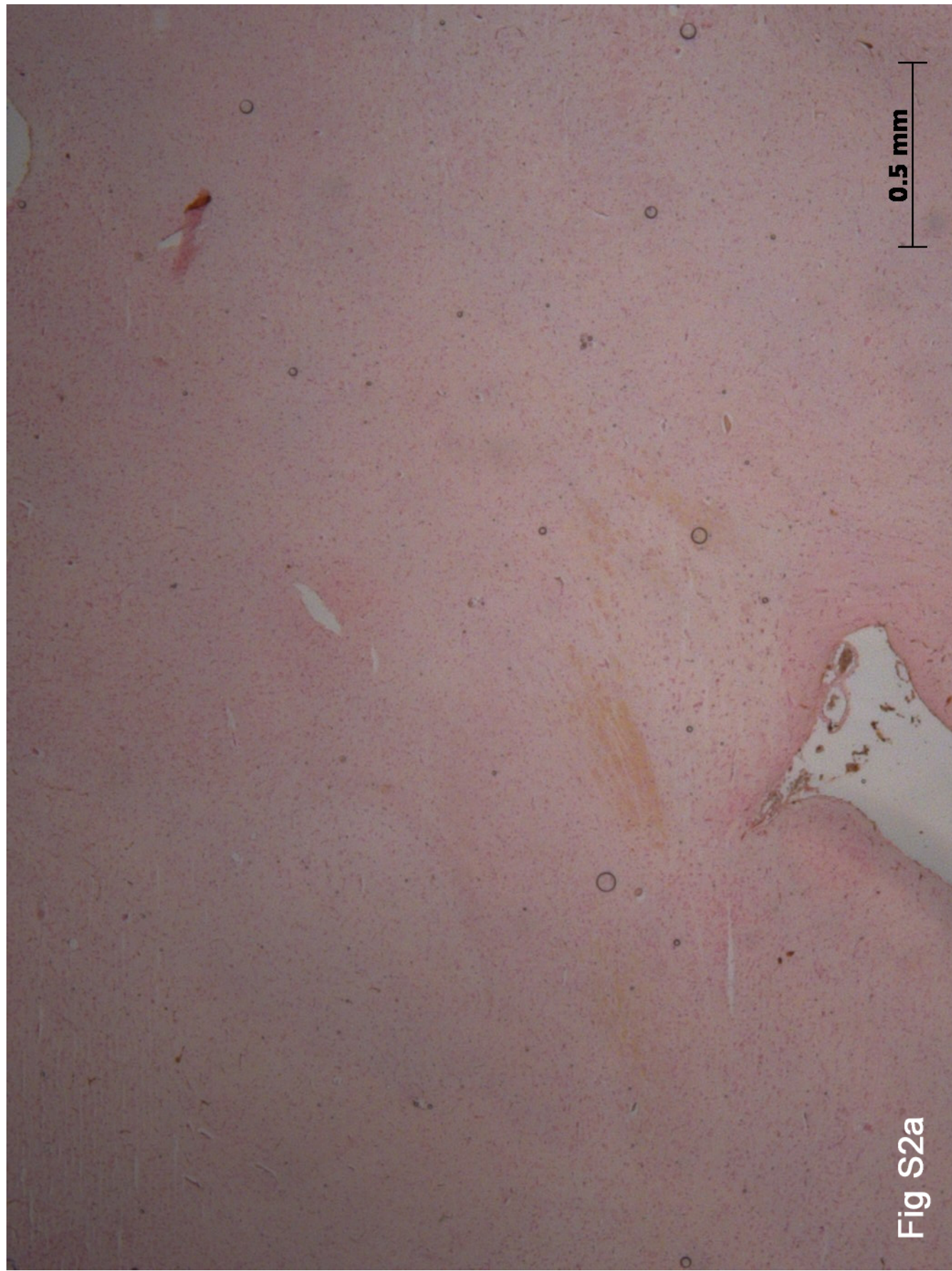

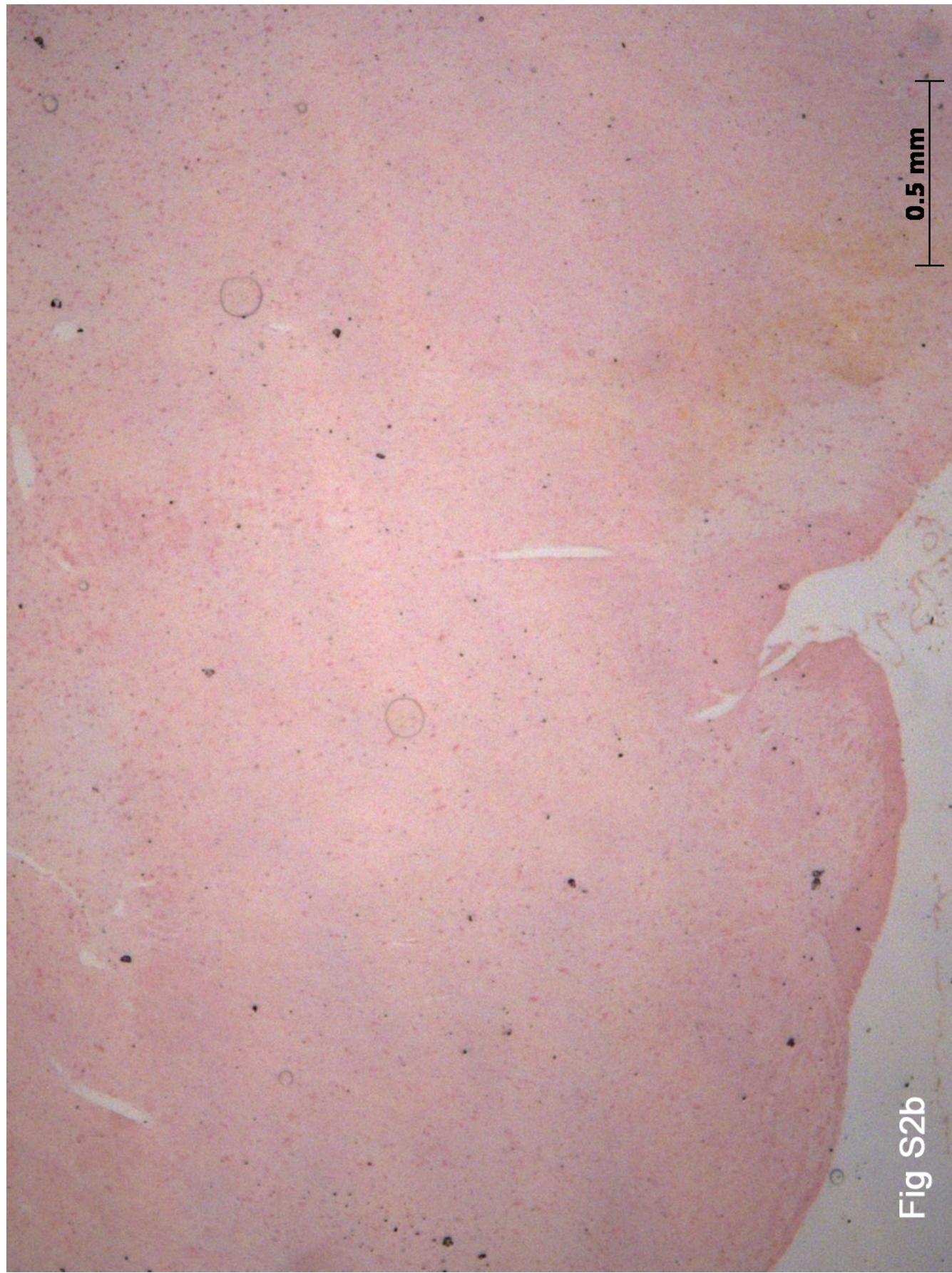

Fig S2b

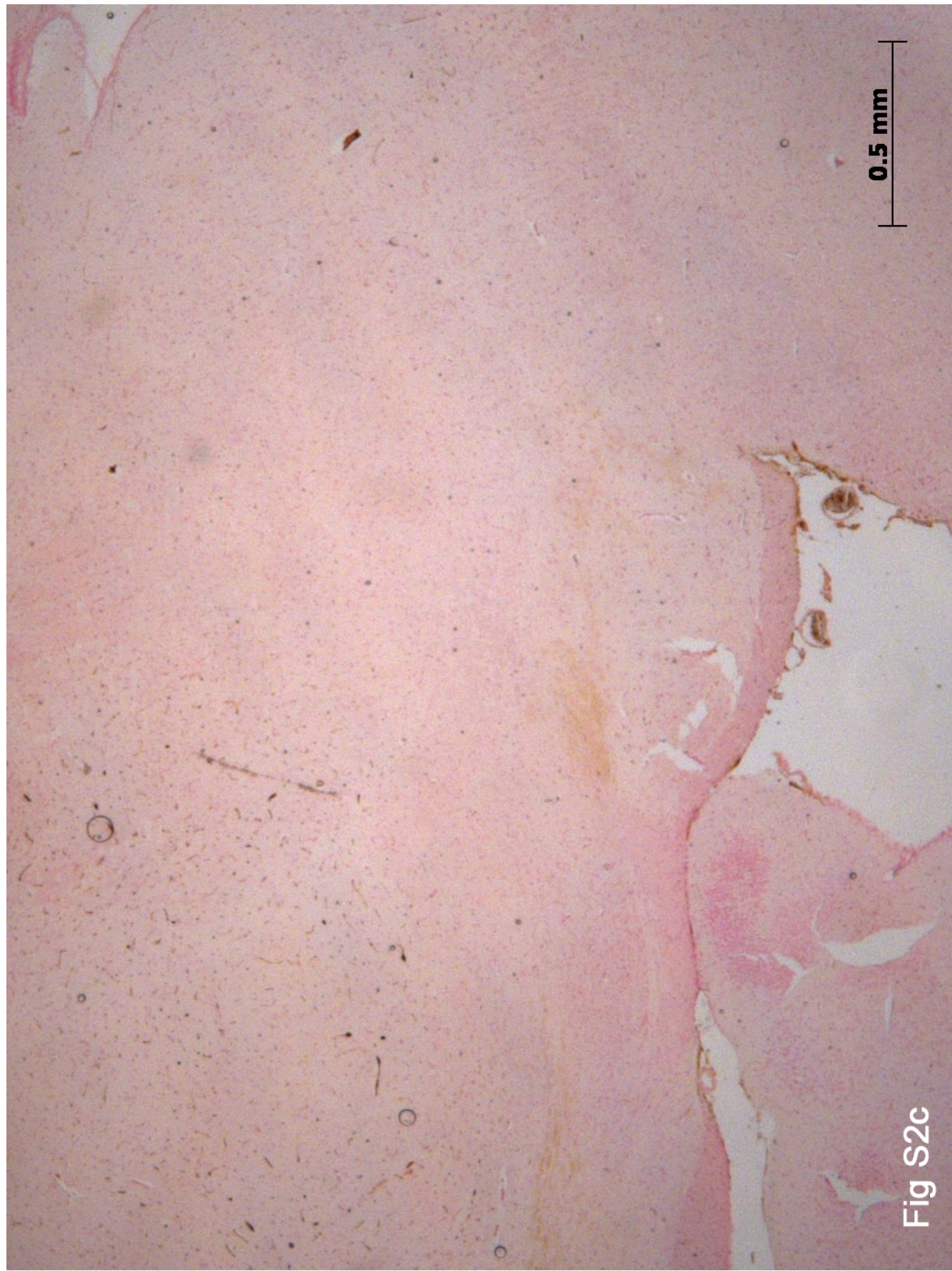

Fig S2c

Supplement: S2 File — a Original immunohistochemical images for DAT for Sham group (magnification scale 2.5 x) b Original immunohistochemical images for DAT for MPTP group (magnification scale 2.5 x) c Original immunohistochemical images for DAT for MPTP-TO901317 group (magnification scale 2.5 x). (PDF) [file pone.0174470.s002.pdf]

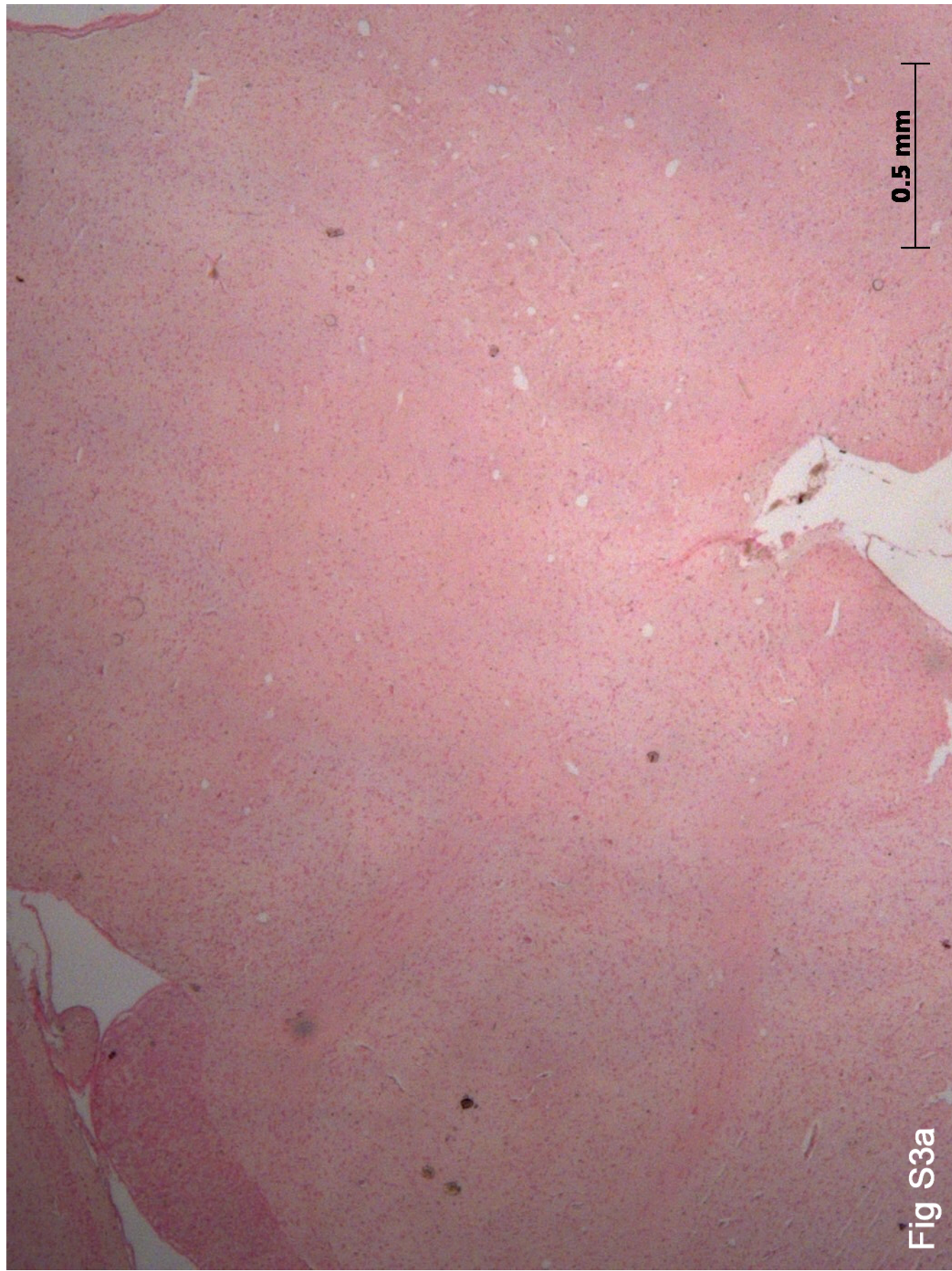

Fig S3a

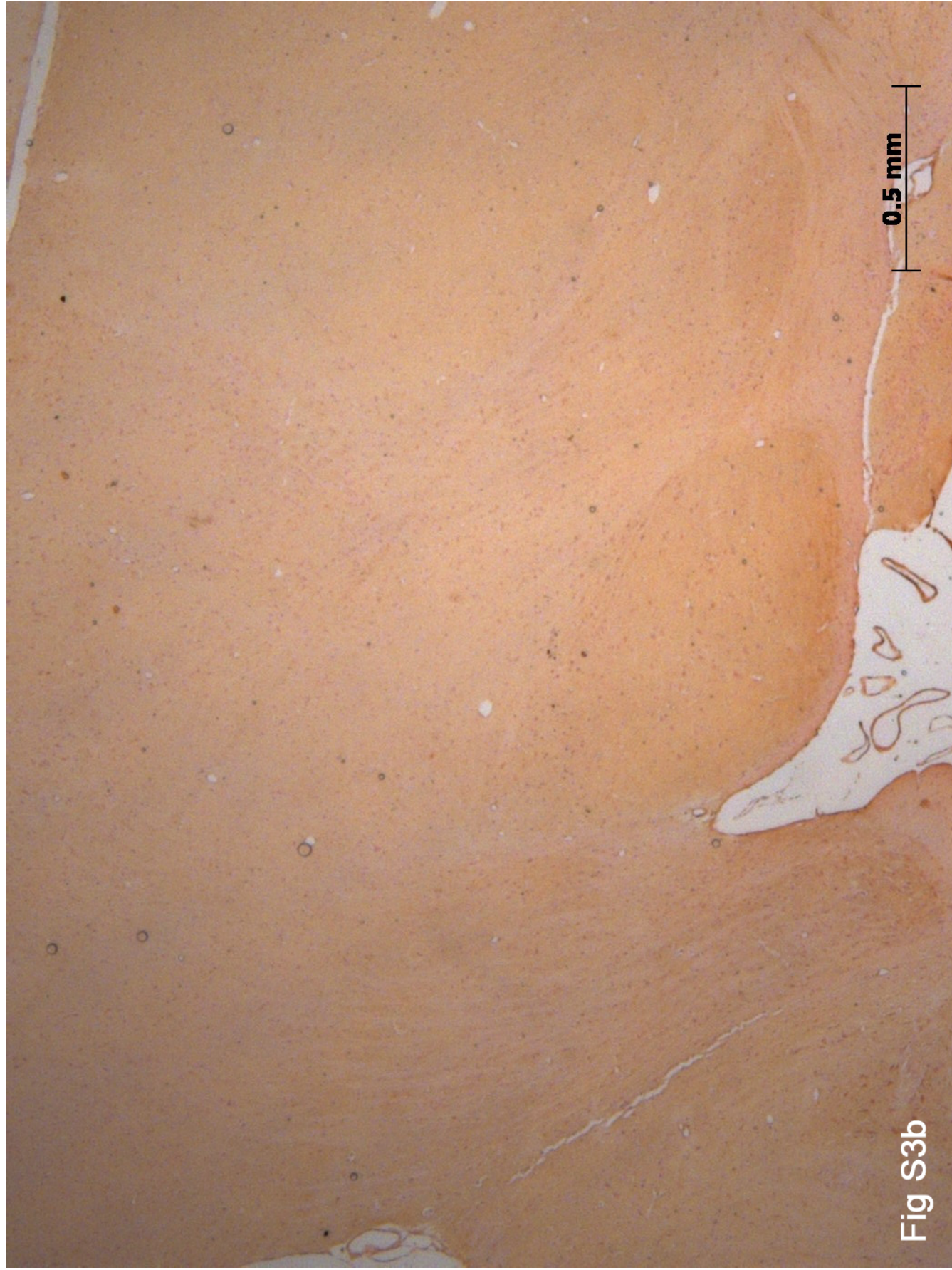

Fig S3b

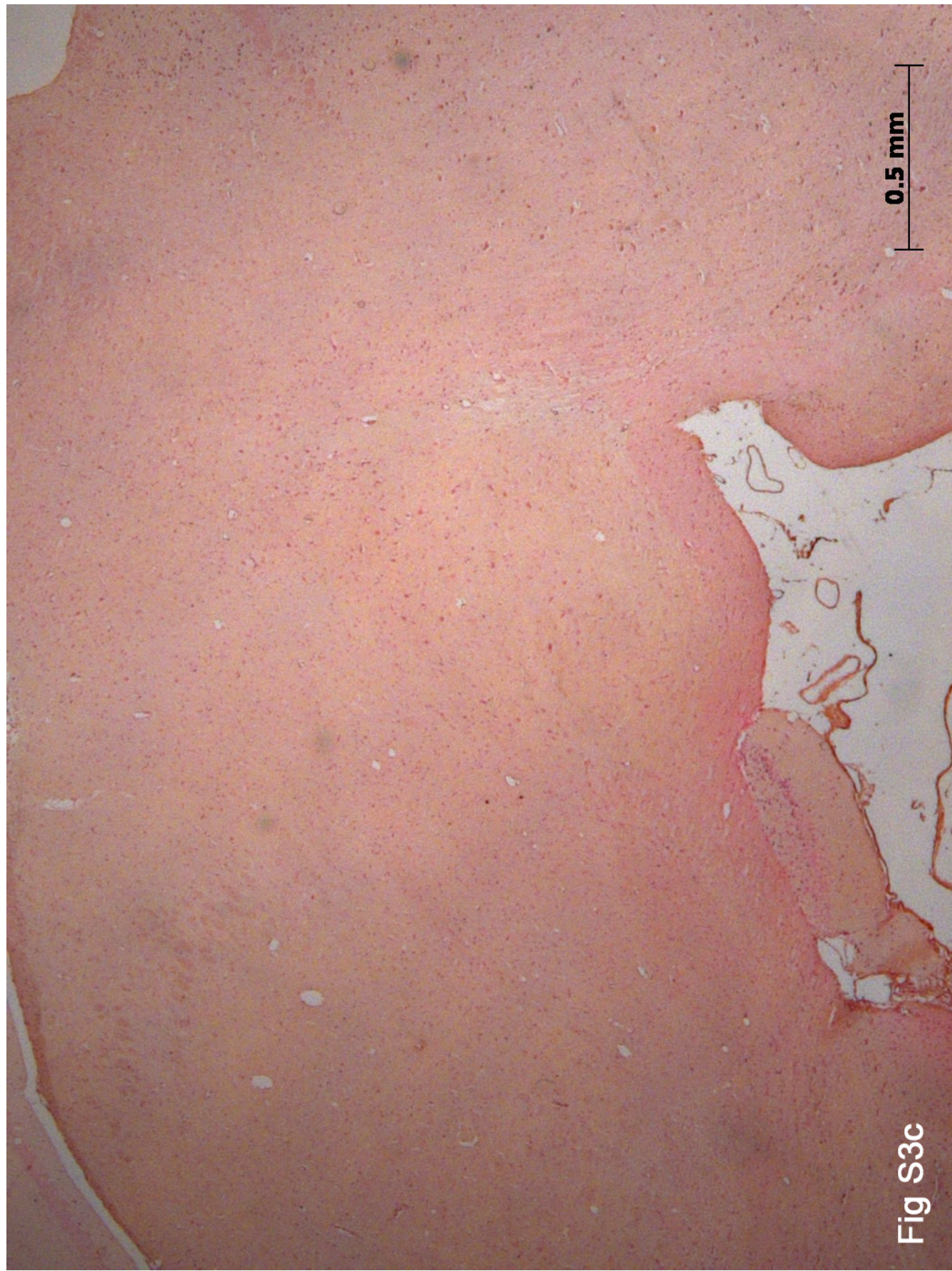

Supplement: S3 File — a Original immunohistochemical images for iNOS for Sham group (magnification scale 2.5 x) b Original immunohistochemical images for iNOS for MPTP group (magnification scale 2.5 x) c. Original immunohistochemical images for iNOS for MPTP-TO901317 group (magnification scale 2.5 x). (PDF) [file pone.0174470.s003.pdf]

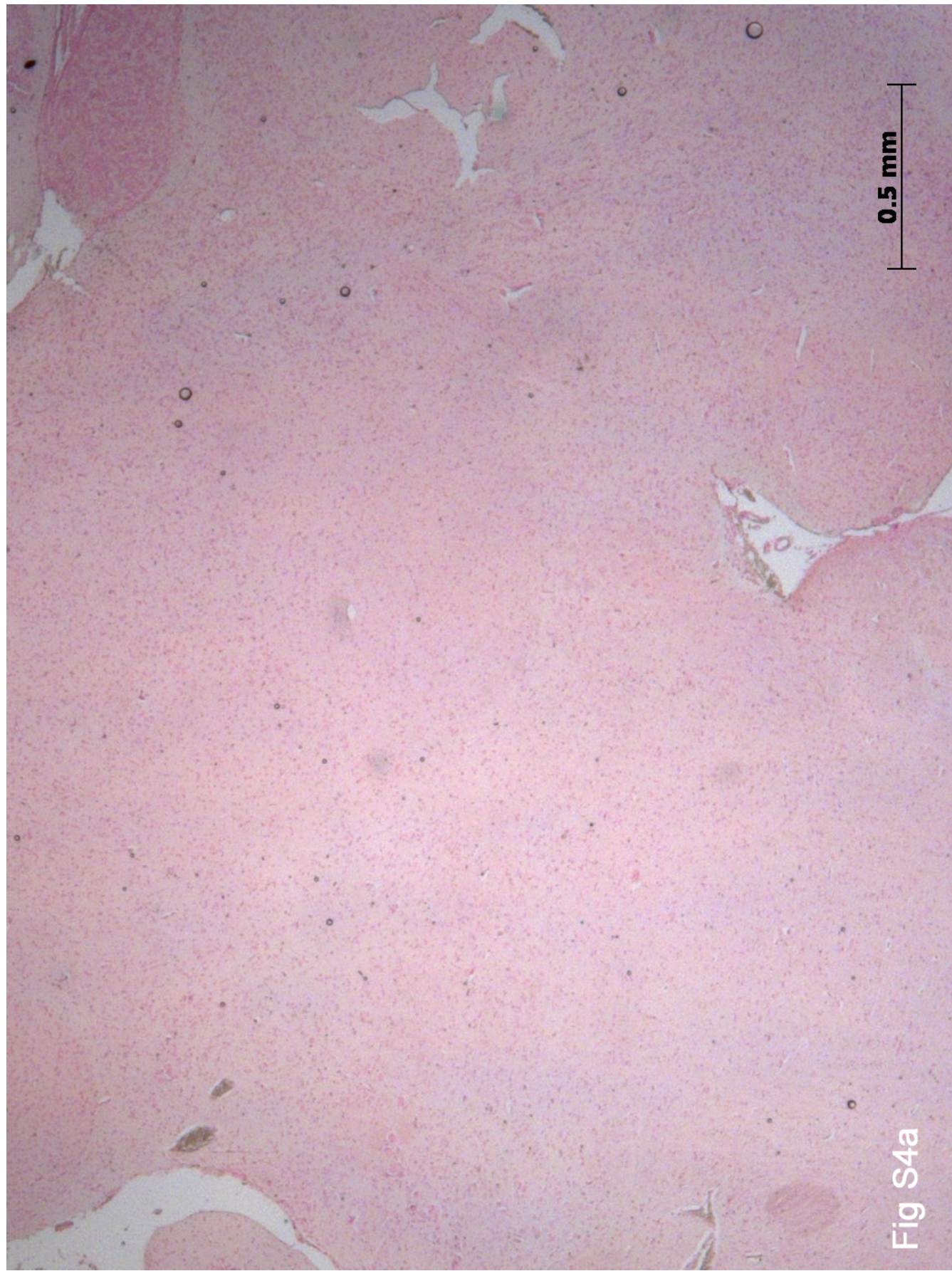

Fig S4a

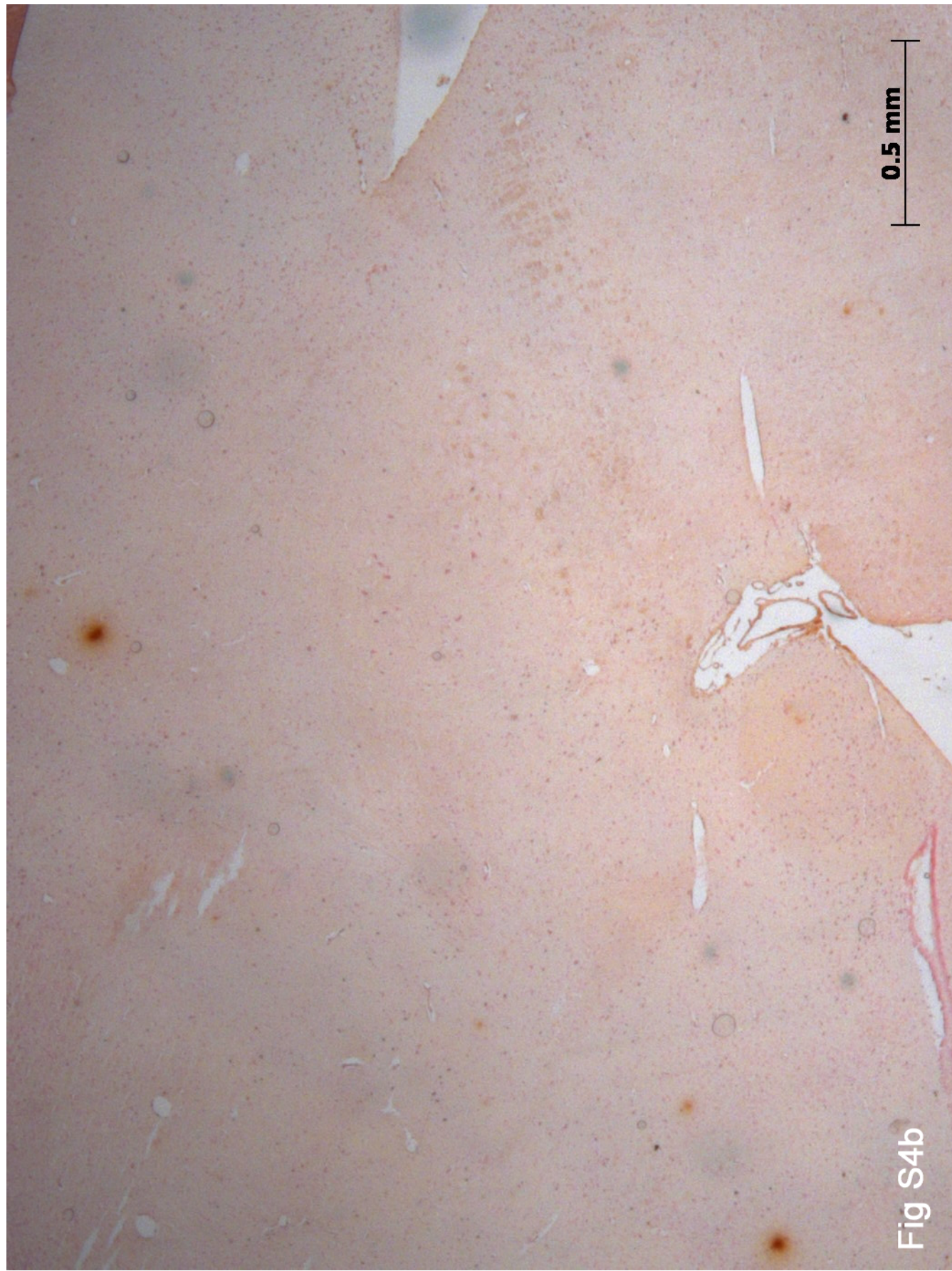

Fig S4b

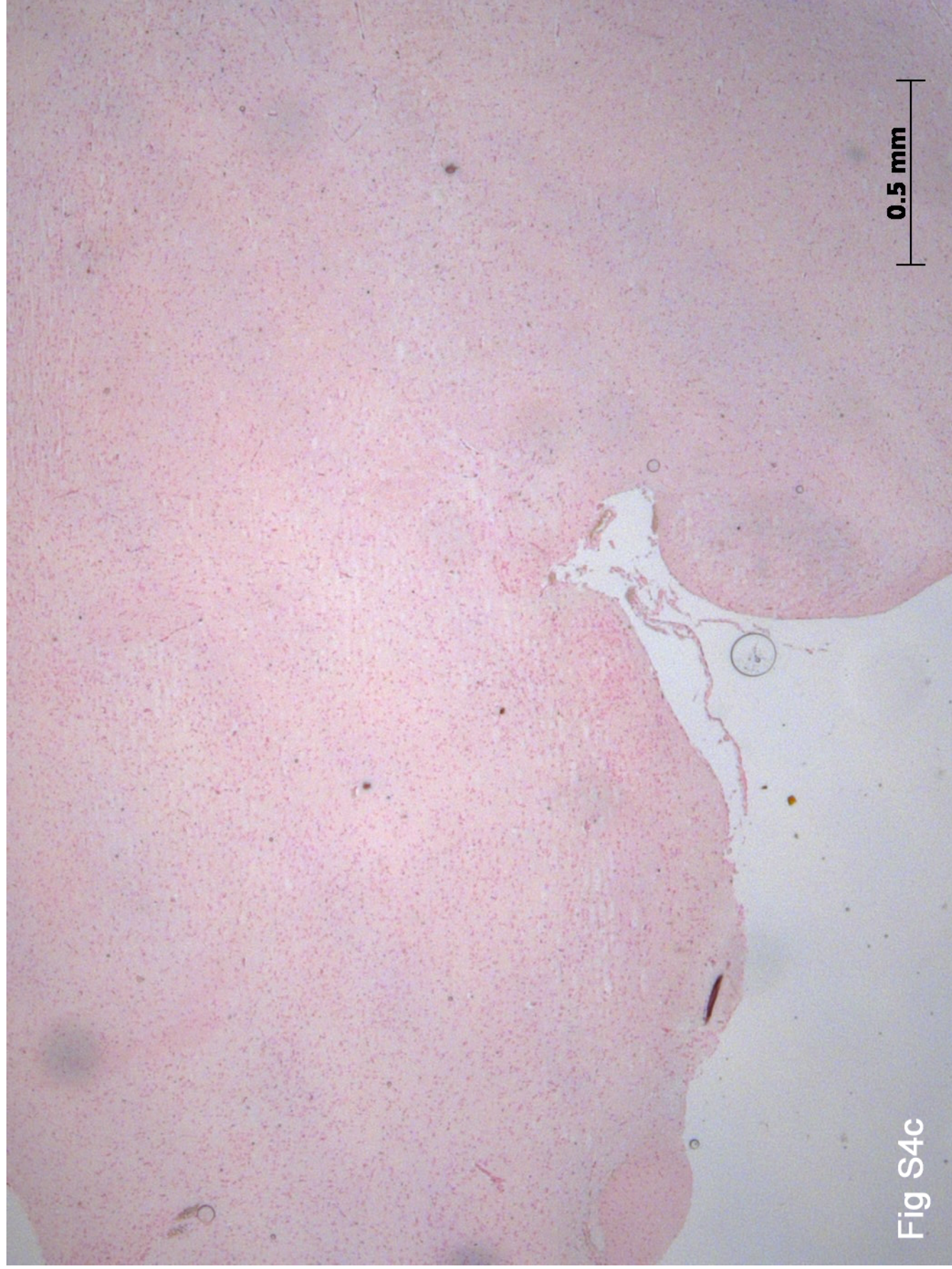

0.5 mm

Fig S4c

Supplement: S4 File — a Original immunohistochemical images for COX-2 for Sham group (magnification scale 2.5 x) b Original immunohistochemical images for COX-2 for MPTP group (magnification scale 2.5 x) c Original immunohistochemical images for COX-2 for MPTP-TO901317 group (magnification scale 2.5 x). (PDF) [file pone.0174470.s004.pdf]

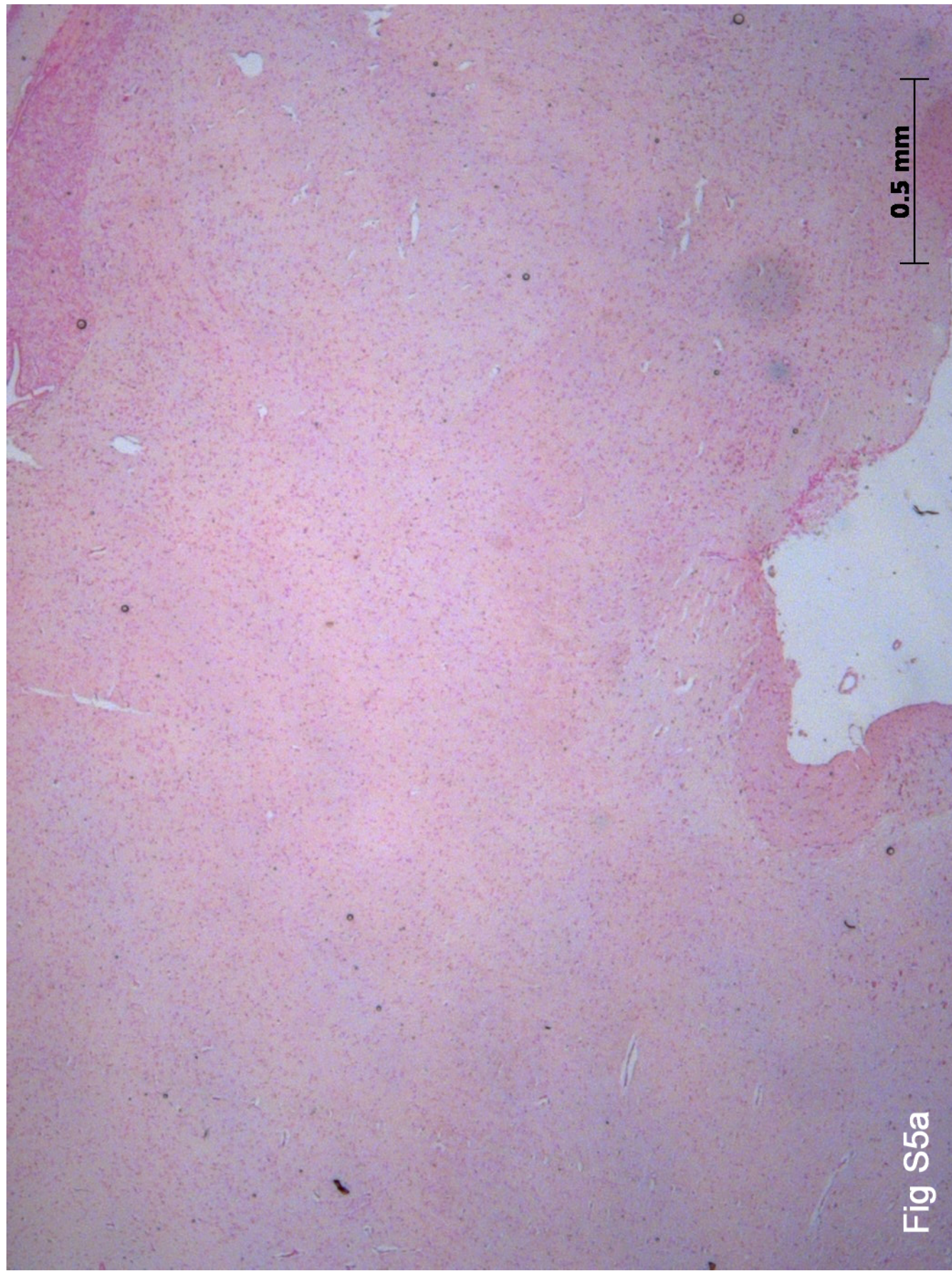

Fig S5a

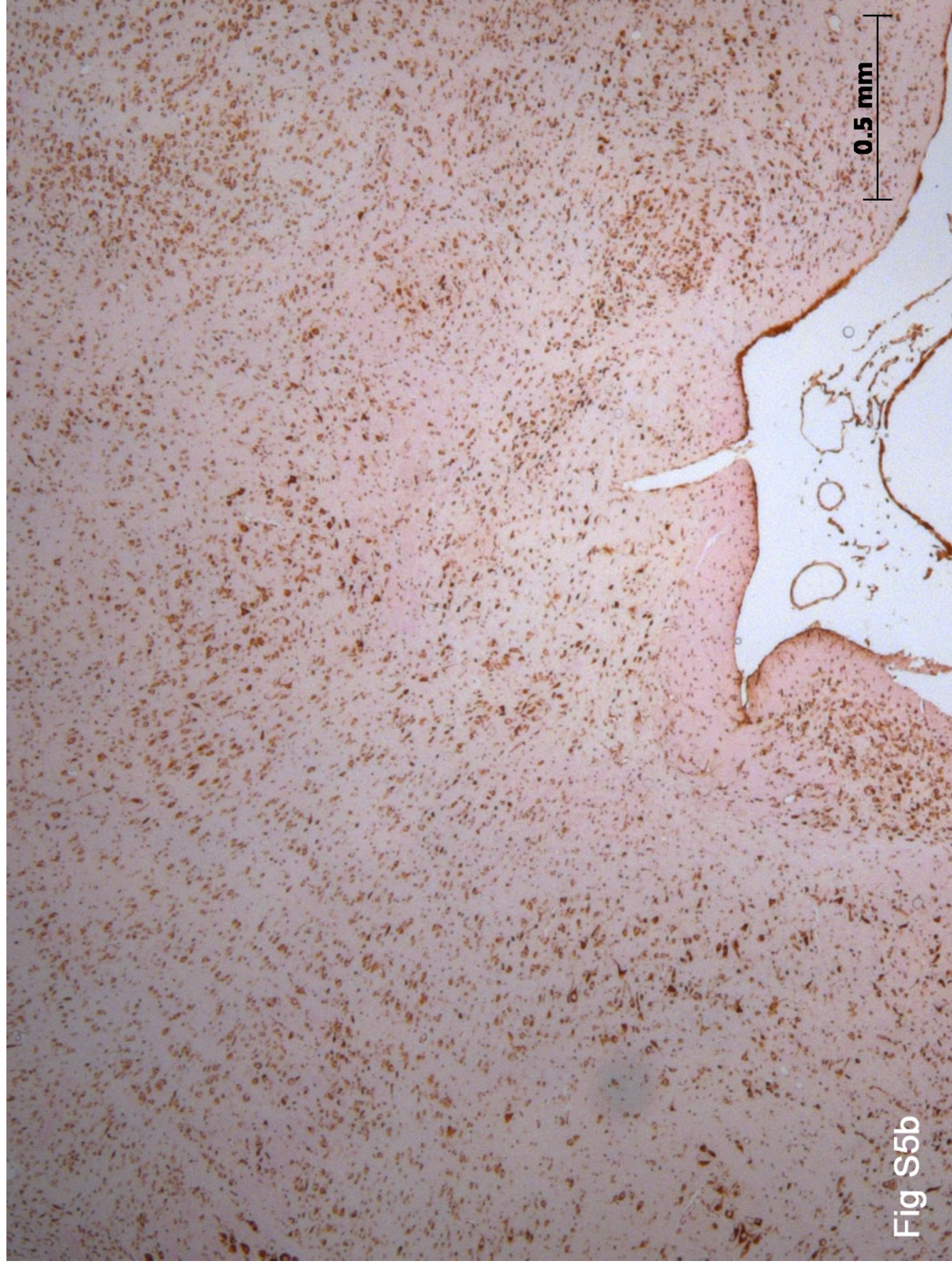

Fig S5b

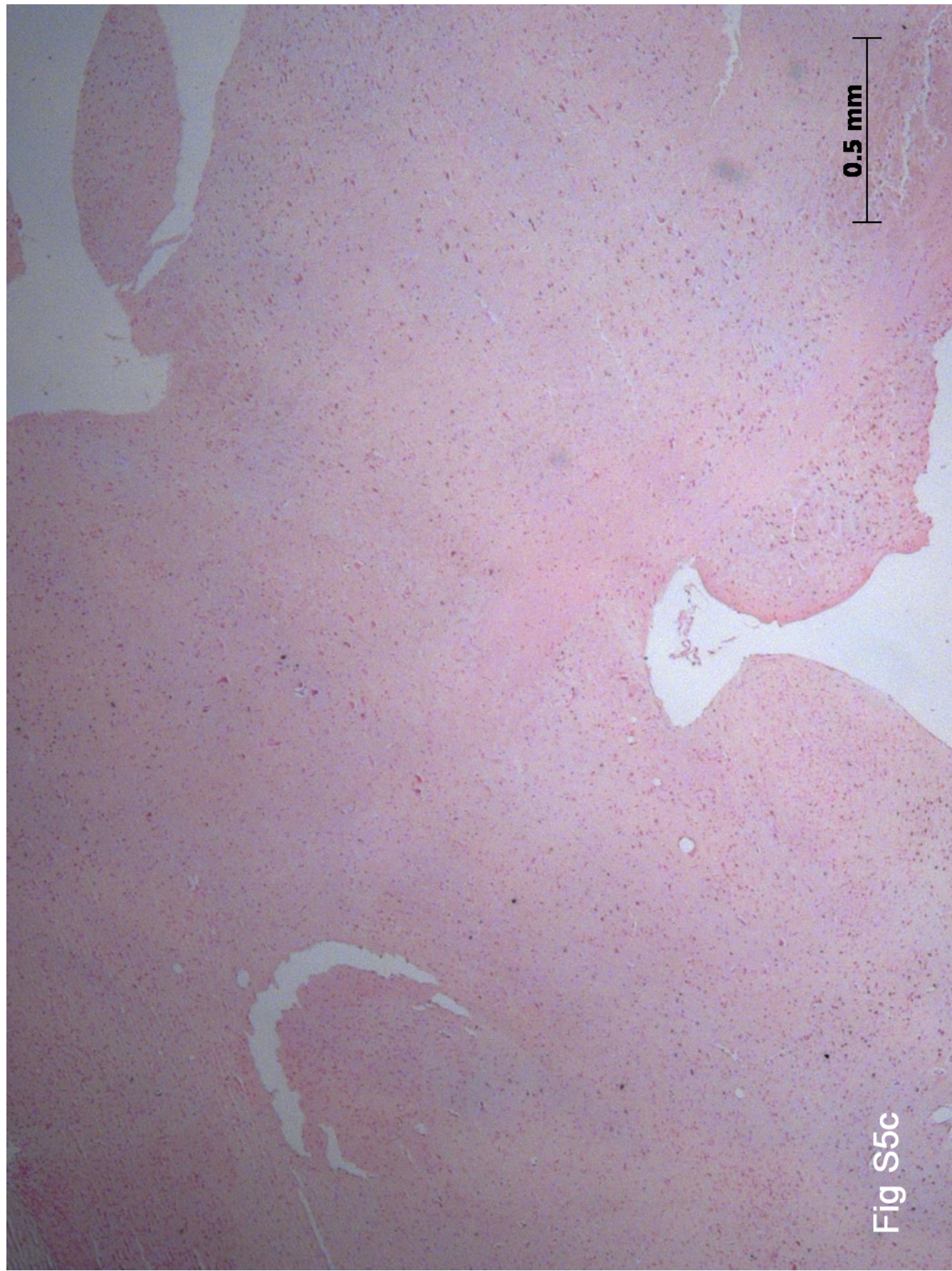

Fig S5c

Supplement: S5 File — a Original immunohistochemical images for BAX for Sham group (magnification scale 2.5 x) b Original immunohistochemical images for BAX for MPTP group (magnification scale 2.5 x) c Original immunohistochemical images for BAX for MPTP-TO901317 group (magnification scale 2.5 x). (PDF) [file pone.0174470.s005.pdf]

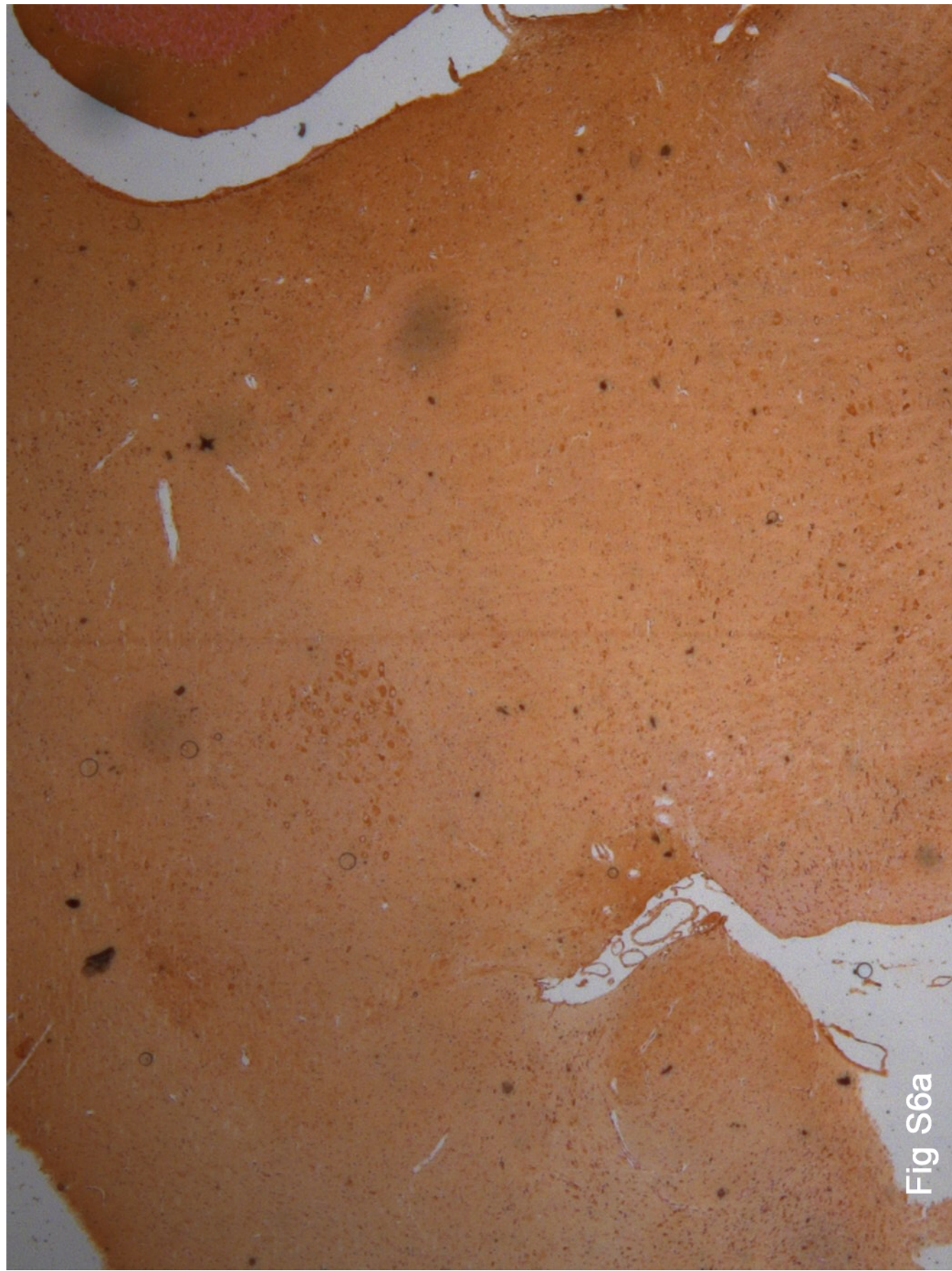

Fig S6a

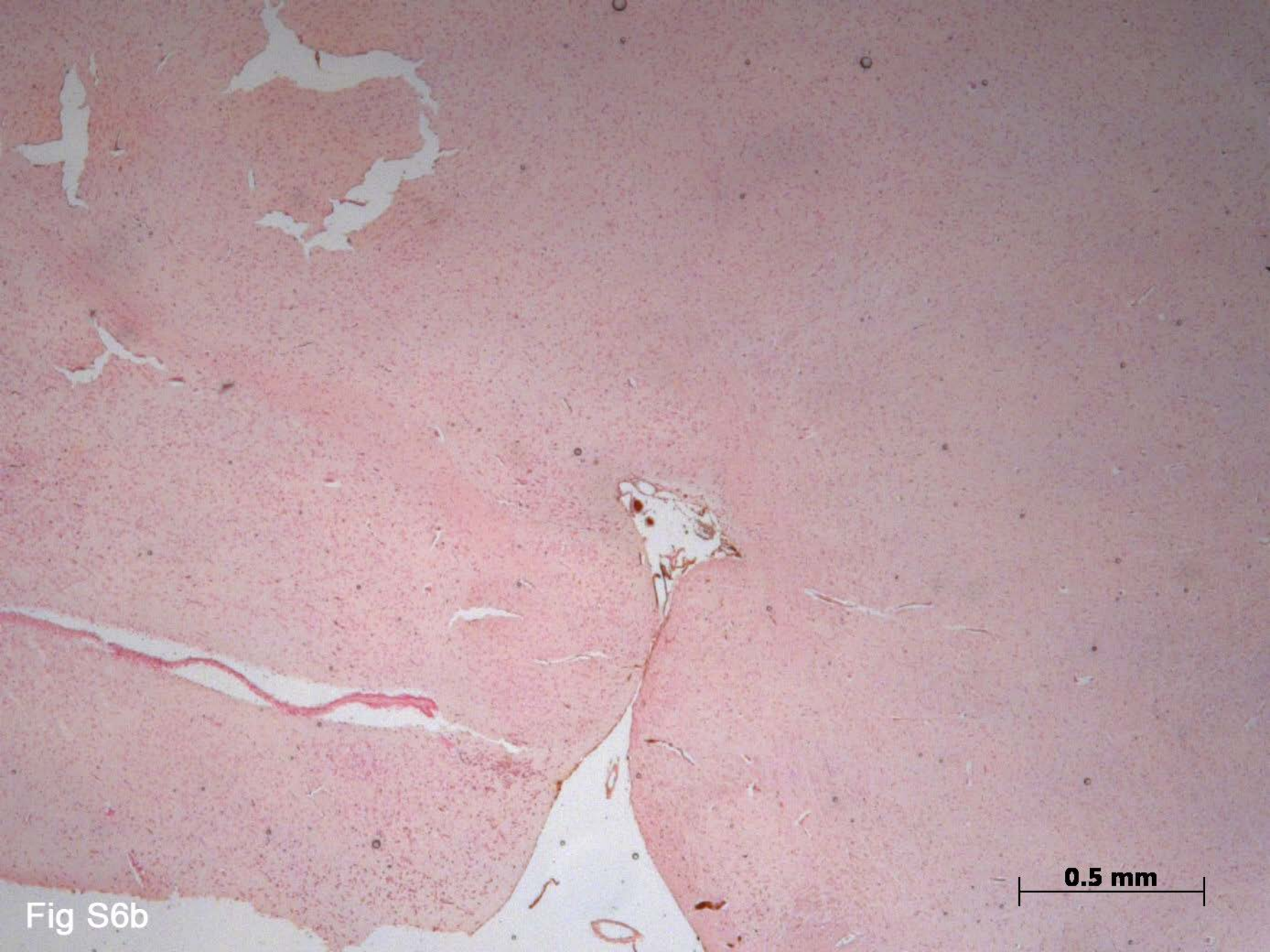

Fig S6b

0.5 mm

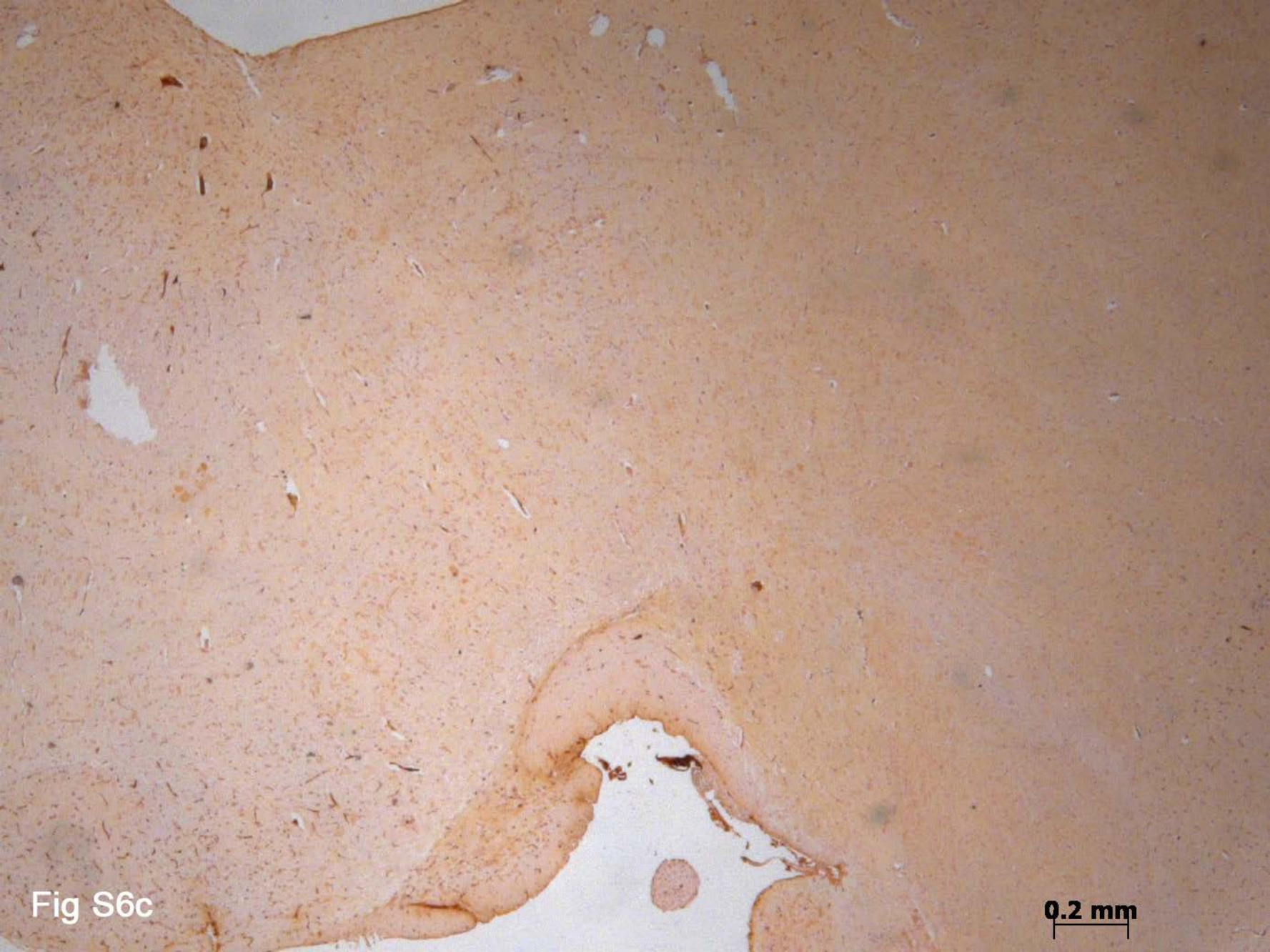

Fig S6c

0.2 mm

Supplement: S6 File — a Original immunohistochemical images for Bcl-2 for Sham group (magnification scale 2.5 x) b Original immunohistochemical images for Bcl-2 for MPTP group (magnification scale 2.5 x) c Original immunohistochemical images for Bcl-2 for MPTP-TO901317 group (magnification scale 2.5 x). (PDF) [file pone.0174470.s006.pdf]

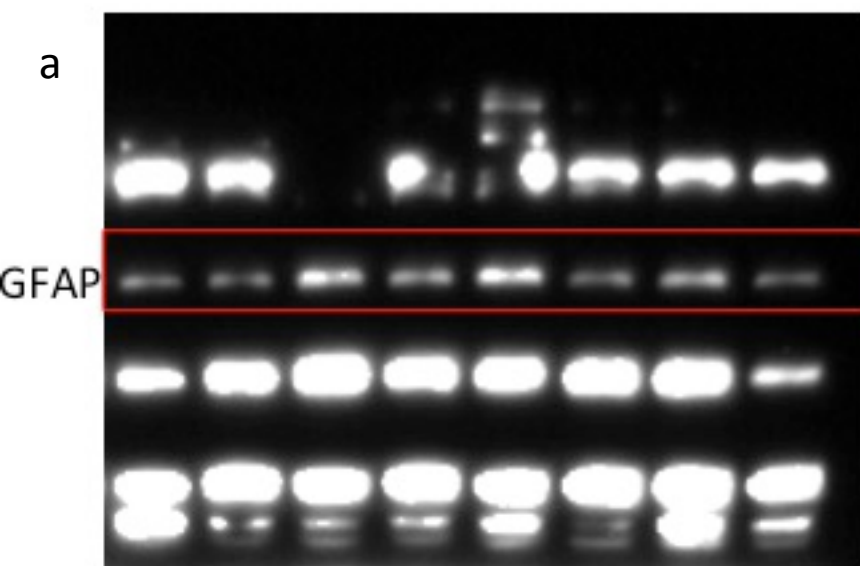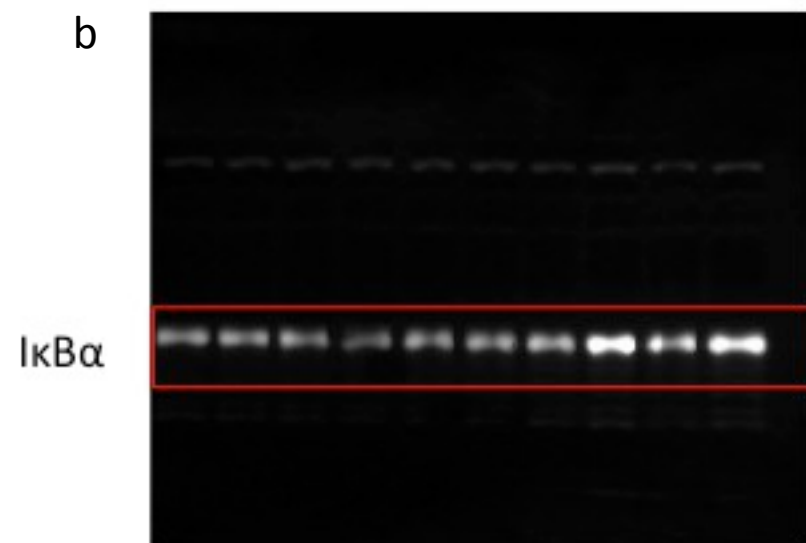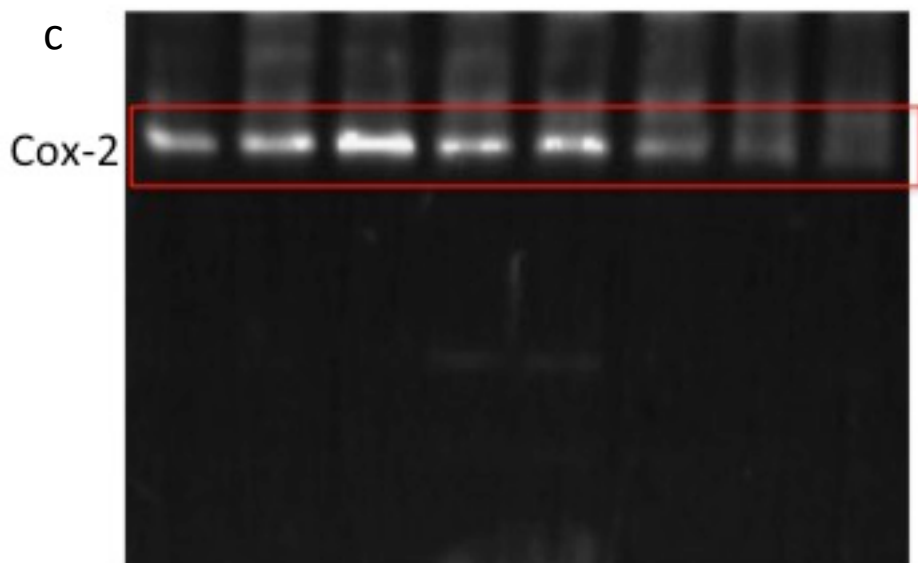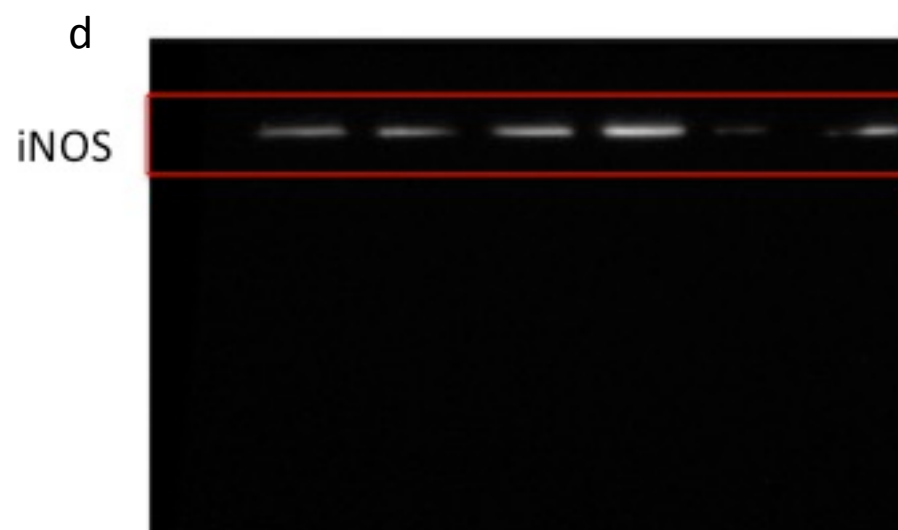

Figure S7

Supplement: S7 File — (PDF) [file pone.0174470.s007.pdf]
